# Clinical application of ^1^H MRS in the human brain at 7T

**DOI:** 10.3389/fnins.2026.1746678

**Published:** 2026-04-30

**Authors:** Graeme A. Keith, Rosemary A. Woodward, Evonne McLennan, Tracey Hopkins, Jon Trinder, Sarah Allwood-Spiers, Likhith Alakandy, Roddy O’Kane, Athanasios Grivas, Emanuela Molinari, Colin O’Leary, George Gorrie, Saif Razvi, Aoife Williamson, Anthony J. Chalmers, William Stewart, Keith W. Muir, David A. Porter, Natasha E. Fullerton

**Affiliations:** 1 School of Psychology and Neuroscience, University of Glasgow, Glasgow, United Kingdom; 2 Glasgow Clinical Research Imaging Facility, NHS Greater Glasgow and Clyde, Glasgow, United Kingdom; 3 Department of Clinical Physics and Bioengineering, NHS Greater Glasgow and Clyde, Glasgow, United Kingdom; 4 Department of Neurosurgery, Institute of Neurological Sciences, Queen Elizabeth University Hospital, NHS Greater Glasgow and Clyde, Glasgow, United Kingdom; 5 Department of Neurology, Institute of Neurosciences, Queen Elizabeth University Hospital, NHS Greater Glasgow and Clyde, Glasgow, United Kingdom; 6 Department of Oncology, Beatson West of Scotland Cancer Centre, Glasgow, United Kingdom; 7 School of Cancer Sciences, University of Glasgow, Glasgow, United Kingdom; 8 Department of Neuropathology, Queen Elizabeth University Hospital, NHS Greater Glasgow and Clyde, Glasgow, United Kingdom; 9 Department of Neurosurgery, Penn Center for Brain Injury and Repair, Perelman School of Medicine, University of Pennsylvania, Philadelphia, PA, United States; 10 School of Cardiovascular and Metabolic Health, University of Glasgow, Glasgow, United Kingdom; 11 Department of Neuroradiology, Institute of Neurological Sciences, Queen Elizabeth University Hospital, NHS Greater Glasgow and Clyde, Glasgow, United Kingdom

**Keywords:** ^1^H MRS, 7T MR safety, 7T MRI, clinical imaging, glioma pathology, imaging biomarkers, neuroimaging, sLASER

## Abstract

Proton magnetic resonance spectroscopy (^1^H MRS) enables non-invasive biochemical sampling of tissues, potentially aiding diagnosis, prognosis and monitoring of various pathologies, while providing novel imaging biomarkers. Ultra-high-field (UHF) imaging at 7 tesla (7T) benefits from improved spectral dispersion due to an increase in chemical shift differences between metabolites, and a higher signal-to-noise ratio (SNR), making ^1^H MRS at 7T a particularly promising diagnostic tool for identifying and separating metabolites not clearly resolved at lower field strengths. However, ^1^H MRS at UHF presents technical challenges related to the short RF wavelength at 7T, resulting in B_1_ transmit field inhomogeneity, and the increased magnetic susceptibility gradients leading to B_0_ field inhomogeneity. Appropriate MRS methods are required to address these issues. In this article, we describe the technical aspects and challenges of ^1^H MRS at 7T, based on the experience in our centre, where single voxel ^1^H MRS has featured prominently in clinical 7T research applications for several years. We present data from six patients with glial tumours, including three who were post-operative, in whom post-surgical metalware affects the specific absorption rate (SAR), along with two patients with neuroinflammatory conditions and two with neurodegenerative diseases. The potential clinical use of ^1^H MRS for these pathologies and its possible integration as a promising biomarker into advanced imaging pathways are discussed.

## 1 Introduction

Proton (^1^H) magnetic resonance spectroscopy (MRS) is a powerful tool for assessing the neurochemical profile of brain tissue and has been applied *in vivo* since the earliest days of MRI (Bottomley, 1997). ^1^H MRS takes advantage of the small differences in the resonance frequencies of hydrogen nuclei situated in different chemical environments, known as chemical shift (*δ*). Besides the dominant water signal from brain tissue, ^1^H nuclei are present in neuron-specific metabolites such as total n-acetyl aspartate (tNAA), choline (tCho), and creatine (tCr); neurotransmitters like glutamate (Glu) and *γ*-aminobutyric acid (GABA); lactate—a product of anaerobic metabolism; the accumulation of disease-specific metabolites such as the oncometabolite 2-hydroxyglutarate (2HG); as well as lipids and contributions from mobile macromolecules (Cudalbu et al., 2021) among many others. In a basic MRS experiment, to measure a spectrum in a single voxel of brain tissue, orthogonal slice-selective radiofrequency (RF) pulses are employed for localisation, such that no spatial encoding during signal acquisition is required. In the absence of a frequency-encoding gradient during the readout, a Fourier transform of the time-domain signal yields a frequency spectrum (Ernst and Anderson, 1966), in which peaks represent different chemical shifts.

As the concentrations of metabolites present in brain tissue are many orders of magnitude lower than the water content of the tissue, it can be challenging to achieve reliable, quantitative results without effective and robust suppression of the water signal. Common chemical shift selective saturation (CHESS)-based methods (Haase et al., 1985) optimised for water suppression include the three-pulse Water Suppression Enhanced through T_1_-effects method (Ogg et al., 1994) and its four-pulse variant for greater water suppression efficiency (Keith and Porter, 2019), as well as variable power radiofrequency pulses with optimised relaxation delays (VAPOR) (Tkac et al., 2021; Tkac et al., 1999), which employs a train of seven or eight pulses for improved insensitivity to RF transmit inhomogeneities and variations in T_1_.

Ultra-high field (UHF) MRI systems (≥7T) have been in use since the early days of this century, and while their use was initially restricted to technical development and advanced neuroscience studies at research sites, there are now over one hundred UHF sites around the world. UHF scanners have been shown to benefit from a supralinear increase in SNR with magnetic field strength (Pohmann et al., 2016; Schick, 2005), where comparable scan protocols and RF receive arrays are employed. This extra SNR allows MR images to be acquired at far higher spatial resolution, unachievable at lower field strengths, or for increased acceleration factors when applying parallel imaging techniques (Griswold et al., 2002; Pruessmann et al., 1999; Wiesinger et al., 2004), or a combination of both. This SNR increase, along with increases in susceptibility differences between tissue types and an increase in T_2_^*^ dephasing, offers potential advantages for many imaging methods, such as susceptibility weighted imaging (SWI) and blood-oxygen level dependent (BOLD) functional imaging (fMRI), when compared with standard clinical field strengths. This has been explored by our group in previous work investigating the clinical translation of 7T imaging in brain pathologies (Keith et al., 2024b) including epilepsy, glial brain tumours, and multiple sclerosis (Fullerton et al., 2024).

Performing MRS at 7T also provides distinct advantages over lower field strengths, including the aforementioned higher SNR, which helps to mitigate signal loss from the shorter T_2_ relaxation values for metabolites of interest at higher field strengths. The balance of these two features of UHF-MRS, higher SNR and shorter T_2_, means that the SNR gain in metabolite signal is likely not in the maximal range seen in some imaging applications (Li et al., 2013). However, given the low concentration of the metabolites of interest, even a modest SNR improvement enhances our ability to detect and quantify these metabolites. In addition to the SNR gain, 7T also provides greater frequency dispersion of metabolite resonances (Tkac et al., 2001). This leads to higher achievable spectral resolution, allowing overlapping resonances, such as the excitatory neurotransmitter glutamate and its precursor glutamine, to be robustly resolved individually and more accurately quantified. Different concentrations of these metabolites have been proposed to exist in oligodendrogliomas compared to astrocytomas (Chawla et al., 2010), with alterations in concentrations reported in many pathologies, such as MS, epilepsy (Petroff et al., 2002), and tumefactive demyelinating lesions (Cianfoni et al., 2007). More accurate demonstration of metabolites at 7T may therefore translate into clinical use and applications.

7T MRS does come with significant challenges, however. The greater inhomogeneity of the B_0_ and B_1_ fields with increasing field strength, which affects all UHF imaging (Koch et al., 2009; Vaughan et al., 2001), has a potentially significant impact on clinical imaging. Clinical imaging relies on signal homogeneity and consistent, high image quality throughout the brain. B_0_ inhomogeneity can cause significant artefact, particularly near air and tissue interfaces, such as in the frontal lobes adjacent to paranasal sinuses and the temporal lobes adjacent to mastoid air cells. This especially impacts pathologies involving these anatomical areas, with frequent involvement of the temporal lobes in patients with glioma and epilepsy, but also in demyelination and other pathologies. This can also be an issue in sequences relying on low-frequency encoding echo-planar imaging, used in functional MRI and diffusion tensor imaging, both highly relevant for pre-operative temporal lobe neuro-oncology and epilepsy imaging. Clear visualisation of the optic radiations, Meyer’s loop, and the Inferior Fronto-occipital Fasciculus are crucial and require optimisation of B_0_ field inhomogeneity.

The impact of the inhomogeneity of the B_0_ and B_1_ fields also affects MRS. The inhomogeneity of the static B_0_ field in the chosen voxel adversely affects the spectral linewidth and can lead to non-Lorentzian lineshapes (Juchem et al., 2021). The linewidth effects can be expressed as a sum of T_2_ line broadening effects, microscopic susceptibility effects within the voxel, and macroscopic effects from susceptibility differences between tissue types (Tkac et al., 2001). The last of these can be accounted for by achieving a good B_0_ shim in the voxel using specialist techniques, resulting in a smaller linewidth and less loss of SNR (Juchem and de Graaf, 2017). The increased spectral linewidth and decreased SNR in anatomically affected areas, such as the temporal lobes, may decrease the sensitivity of spectroscopy, with spectral dispersion of metabolites lost at post-processing.

The inhomogeneity in the radiofrequency transmit field (B_1_^+^), caused by interactions with the decreased wavelength RF energy applied at 7T, leads to highly variable flip angles across the brain. In principle, this can be mitigated in the application of single-voxel MRS, as once the voxel has been planned, the reference voltage or flip angles of the RF pulses can be adjusted so that the desired flip angle is achieved within the voxel. This can be an effective strategy for rough B_1_^+^ scaling, but it adds an additional optimisation step. In brain regions with inherently poor B_1_^+^, the maximum achievable flip angle may be restricted by specific absorption rate (SAR) limits, which measure the RF power deposited in tissue and scale with B_0_^2^ (van der Zwaag et al., 2016). This may require further changes to pulse timings or repetition time (TR) to successfully run the sequence within SAR limits. In imaging applications at UHF, much work has been done to mitigate B_1_^+^ field inhomogeneity effects with the application of parallel transmit (pTx) methods (Katscher et al., 2003; Zhu, 2004). These methods use novel RF coils with multiple transmit elements (Adriany et al., 2005; Williams et al., 2021), which can be driven independently to create a more homogeneous transmit field and therefore reduce flip angle variation across the field of view. This can be achieved by varying the amplitude and phase of the RF pulses at each transmit element—an approach known as static pTx, RF shimming, or B_1_^+^ shimming (Mao et al., 2006), or by further varying the pulse shape transmitted by each element, known as dynamic pTx (Grissom et al., 2006; Katscher et al., 2003). These methods have been used to improve imaging techniques, such as T_1_-weighted anatomical sequences (Cloos et al., 2012), spin-echo-based sequences (Malik et al., 2015; Beqiri et al., 2018), and diffusion imaging (Ding et al., 2024; Wu et al., 2018), and some work has been done to apply pTx to both single-voxel (Berrington et al., 2021; Emir et al., 2012) and multi-voxel MRS methods (Keith et al., 2024a; Boer et al., 2012). Achieving a homogeneous B_1_^+^ field is particularly important for clinical imaging, where areas of signal dropout, most noticeably occurring in the temporal lobes and posterior fossa at 7T, could make the detection of pathology, such as demyelinating lesions in MS, characteristically found adjacent to the temporal horns, challenging.

More specific to MRS at higher fields than the universal issues described above is the problem of chemical shift displacement error (CSDE). Due to the frequency dispersion of metabolite resonances with increasing field strength, the frequency difference between two metabolite peaks in Hz is greater at 7T than at lower field strengths. While this greater spectral resolution allows for more peaks to be reliably resolved, issues arise due to the limited transmit bandwidth of the RF pulses used for voxel localisation (Henning et al., 2008). This leads to a spatial mismatch in the signals from metabolites with different resonance frequencies, equal to the quotient of the frequency difference in Hz to the bandwidth of the localisation pulse. While early efforts to counter the effects of CSDE at lower field strengths focused on expanding the excited volume beyond the voxel of interest and then employing very selective saturation (VSS) pulses to suppress signal from outside the volume (Tran et al., 2000), most higher field single-voxel MRS applications use an adiabatic pulse-based sequence, such as Semi-adiabatic Localization by Adiabatic SElective Refocusing (sLASER) (Scheenen et al., 2008).

Adiabatic pulses are both amplitude- and frequency-modulated pulses (Tannús and Garwood, 1996), and thus can be used to achieve far higher transmit bandwidths than standard RF pulses, with greater insensitivity to B_1_^+^ inhomogeneity. The sLASER sequence employs slice-selective excitation, often using an asymmetric sinc pulse for improved slice profile, followed by two pairs of adiabatic inversions, such as hyperbolic secant pulses, in orthogonal directions to further define the chosen voxel, with far lower CSDE than if conventional pulses are used. Pairs of inversion pulses must be used in each direction, as a single adiabatic inversion will leave a nonlinear phase variation across the slice, which is effectively compensated for by the second pulse (Conolly et al., 1991). The sLASER sequence has been recommended for advanced MRS applications at fields of 3T and above (Öz et al., 2021; Wilson et al., 2019).

With newer treatments becoming available, the management of many neurological conditions has changed dramatically, with a shift towards precision medicine (Chitnis and Prat, 2020; Comabella et al., 2016; Morita-Sherman et al., 2025; Roncevic et al., 2023). Personalised diagnostic approaches are required to facilitate patient-centred treatment; however, these remain often still an aspiration (Mowforth et al., 2023) due to a lack of suitable diagnostic biomarkers, especially non-invasive imaging biomarkers. Neuroimaging at 7T, with its high-resolution imaging, high SNR, and increased susceptibility-related dephasing, potentially offers more precise and earlier diagnosis for neurodegenerative, neuroinflammatory, and neuro-oncological conditions (Chalia et al., 2021; de Vries et al., 2025; Duzel et al., 2021; Fullerton et al., 2024) with the improved MRS capabilities of 7T promising to provide novel imaging biomarkers (Okada et al., 2022) that facilitate precision medicine approaches.

Regulatory approval of 7T MRI systems, including FDA approval in the USA and CE marking in Europe in 2017, has opened the door for 7T MRI to become a clinical diagnostic tool. The number of 7T sites has been increasing steadily since regulatory approvals; however, clinical use is still limited due to the discussed technical challenges of 7T MRI. An additional factor limiting the uptake of 7T MRI across more clinical sites is the high financial outlay required for installation, maintenance, and day-to-day running of these systems.

The non-invasive biochemical sampling of MRS is particularly useful in neuroimaging, where obtaining tissue for pathology requires major surgical intervention with associated risks. At 3T, the clinical use of MRS is mostly restricted to neuro-oncology applications, as an adjuvant to structural imaging, to aid pre-operative characterisation of glial brain tumours (Padelli et al., 2022), though its potential has not yet been fully exploited at routine field strengths, let alone at UHF. In 2021, the revised World Health Organization (WHO) classification of tumours of the central nervous system emphasised the importance of molecular phenotyping alongside morphological assessment in tumour diagnosis, with the “holy grail” of tumour imaging and MRS being the identification of possible imaging biomarkers for molecularly defined tumour subtypes (Louis et al., 2021). Other brain pathologies may also be associated with recognised gene associations, such as MND, or alterations in metabolism, stimulating a search for imaging biomarkers to accelerate and advance diagnosis (Mazón et al., 2018). Improved characterisation of metabolites *in vivo* using spectroscopy at 7T could play an important role in developing imaging biomarkers for neurological pathologies.

In this work, we describe potential and promising clinical applications of ^1^H MRS at UHF and discuss MRS techniques and specific challenges at 7T MRI. We present results from ten patients scanned at our centre, with a range of pathologies, to determine whether 7T MRS can reveal disease-specific metabolic signatures, which may improve diagnostic accuracy and inform patient management across diverse neurological conditions.

## 2 Materials and methods

MRS techniques at 7T were optimised in healthy volunteers under local ethics approvals. Patients with various neurological and neurosurgical conditions were scanned as part of different research studies with national (West of Scotland Research Ethics Committee, REC Ref 18/WS/0141) and local Glasgow Clinical Research Imaging Facilities (CRIF) ethics approvals (GN22NE122 and GN23NE233). Pathologies for which single voxel ^1^H MRS was performed included pre- and post-operative low- and high-grade brain tumours, multiple sclerosis (MS), progressive multifocal leukoencephalopathy (PML), motor neuron disease (MND), and epilepsy, with ten examples presented in this article. Data are included from a completed study comparing advanced MRI techniques at 3T and 7T in patients with a range of vascular, inflammatory, neurodegenerative, and malignant neurological conditions; an ongoing study assessing single voxel MRS metabolite alterations in patients with a pre-operative low-grade glioma over consecutive scans; and an ongoing study evaluating 7T imaging and single voxel MRS in patients with glioblastoma post-radiotherapy for early detection and differentiation of disease progression and radiation necrosis. In all studies, voxel placement was guided by a neuroradiologist. In most cases, MRS in a control voxel was not acquired due to time constraints.

All imaging and MRS data were acquired on a 7T MAGNETOM Terra scanner (VE12U_SP01, Siemens Healthcare, Erlangen, Germany) with a 32-channel receive ^1^H RF quadrature head coil (Nova Medical, Wilmington, MA, USA).

### 2.1 MRS acquisition

MRS was performed with a single voxel sLASER sequence, due to its robust voxel localisation and reduced sensitivity to chemical shift displacement error (CSDE). sLASER is the recommended sequence for high and ultra-high field MRS (Öz et al., 2021). Data were acquired with voxel sizes of 1.8–8.4 cm^3^, a repetition time (TR) of 5,000 ms, TE 26–40 ms, 64 averages with all single-shot transients saved, a spectral width of 6 kHz with 2048 complex points, and a transmit frequency offset of −1.7 ppm relative to water. VAPOR water suppression and 3D outer-volume suppression (OVS) were applied, and unsuppressed water spectra were also acquired with and without gradients for quantification and eddy current correction purposes, respectively. The Fast Automatic Shim Technique using Echo-planar Signal readouT for Mapping Along Projections (FAST(EST)MAP) shimming tool (Gruetter, 1993) was used prior to the acquisition to improve B_0_ field homogeneity in the voxel of interest, with an unsuppressed water peak linewidth of ≤13 Hz deemed an acceptable shim (Juchem et al., 2021). FASTESTMAP was typically run for five iterations: first, a multi-echo acquisition to optimise the linear shim terms only, using three projections, followed by three multi-echo iterations of optimisation with six projections for all shim terms up to and including second order, and finally a single-echo acquisition for linear terms only with six projections. For most regions, this procedure was sufficient to provide an acceptable shim; however, where the unsuppressed water linewidth was >13 Hz, the procedure was repeated with more iterations of the optimisation for all shim terms. Optimisations were performed for the flip angle and water suppression train (flip angle and timing) prior to the acquisition. Short echo times were chosen to minimise signal loss from T_2_ decay, enabling the greatest number of metabolites to be identified.

### 2.2 MRS processing and analysis

Preprocessing of the MRS data was performed using the Magnetic Resonance spectral processing and analysis (MRspa) package (Deelchand, University of Minnesota) in MATLAB (R2022b, Mathworks, Natick, MA). Corrections were performed for eddy current effects using the water reference scan acquired with the water suppression spoiler gradients turned on. Frequency and phase instabilities during acquisition, caused by patient motion and scanner drift, were corrected using cross-correlation to minimise the frequency and phase differences between individual transients. The resulting spectra were then fitted for quantitative results in LCModel (Provencher, 1993) using bespoke basis sets containing 21 metabolites and measured macromolecule components. The included metabolites were alanine (Ala), aspartate (Asp), ascorbic acid (Asc), creatine (Cr), cystathionine (Cys), *γ*-aminobutyric acid (GABA), glucose (Glc), glutamine (Gln), glutamate (Glu), glycerophosphorylcholine (GPC), glutathione (GSH), inositol (Ins), scyllo-inositol (sIns), lactate (Lac), phosphocreatine (PCr), phosphorylcholine (PCho), phosphorylethalonamine (PE), N-acetyl-aspartate (NAA), N-acetyl-aspartyl-glutamate (NAAG), taurine (Tau), and 2-hydroxyglutarate (2HG).

All MR spectra are presented according to an expert consensus on minimum reporting standards for *in vivo* MRS (MRSinMRS) (Lin et al., 2021).

### 2.3 Imaging

All patients were scanned under tailored, disease-specific 7T clinical research imaging protocols, as determined by Neuroradiology. Pulse sequence parameters for the images shown are presented in Table 1. Imaging included a T_1_-weighted, two readout variant of the Magnetisation-Prepared RApid Gradient Echo sequence (MP2RAGE (Marques et al., 2010)), which provides greater robustness to the influence of B_1_ inhomogeneities prevalent at higher fields than standard MPRAGE (Mugler and Brookeman, 1991). T_2_-weighted imaging was performed with Turbo Spin-Echo (TSE), along with T_2_-FLuid Attenuated Inversion Recovery (FLAIR). Susceptibility-Weighted Imaging (SWI) was used to visualise the venous system, blood products and mineral deposition, and multi-shot Diffusion Weighted Imaging (DWI) was performed with REadout Segmentation Of Long Variable Echo (RESOLVE (Porter and Heidemann, 2009)) for reduced sensitivity to susceptibility artefacts. Some tumour patients were moved directly from a 3T MRI to the 7T MRI and had MP2RAGE imaging acquired shortly after intravenous gadolinium was administered during their routine clinical 3T MRI scan. In these cases, the gadolinium-based contrast agents used included gadoterate meglumine at a dose of 0.2 mmol/kg of body weight, and gadobutrol at 0.1 mmol/kg of body weight.

**Table 1:** Parameters for 7T structural sequences.

Pulse sequence	TR/TE (ms)	Image resolution (mm^3^)	Image matrix	Slices	Acceleration	BW/px (Hz)	Other	Acq. time (mm:ss)
MP2RAGE	5000/1.94	0.8*0.8*0.8	320*320	208	GRAPPA 3	490	–	08:27
Axial TSE	9000/58	0.4*0.4*3.0	512*384	39	GRAPPA 2	287	Turbo factor 9	03:38
Coronal TSE	9000/58	0.4*0.4*2.0	512*384	35	GRAPPA 2	287	Turbo factor 9	03:38
FLAIR	12,820/59	0.5*0.5*3.0	448*336	39	GRAPPA 2	286	Turbo factor 9	04:31
SWI	21/14	0.2*0.2*1.5	896*728	72	GRAPPA 3	210	–	07:29
RESOLVE-DWI	4890/58	1.0*1.0*3.0	224*224	34	GRAPPA 3	698	b-Value 0/1000 s/mm^2^	03:42

### 2.4 SNR and linewidth

Values for the SNR and linewidth of all spectra were recorded. The SNR was calculated as the ratio of the maximum signal, in the range of 0.5 to 4.2 ppm, minus the baseline to two times the root mean square of residuals, as reported by LCModel. The linewidth was calculated at the scanner by fitting the unsuppressed water peak and measuring the full width at half maximum (FWHM).

### 2.5 Safety

In the case of the post-operative scans conducted in patients with primary brain tumours, the patients had undergone a craniotomy either for extended biopsy or debulking of the glioma, with the craniotomy plate held in place post-operatively via small metal fixation plates. This metalware within the subcutaneous tissues of the skull could cause heating of tissue and potentially a local burn, so an appropriate SAR limit must be considered.

Each participant had small titanium cranial fixation plates to secure the bone flap (Zimmer Biomet, Jacksonville, FL). These were approved for scanning at 7T under a local institutional risk assessment, informed by other studies (Chen et al., 2016; Kraff et al., 2013; Sammet et al., 2013; Shaffer et al., 2023). Initially, patients were deemed eligible for a 7T scan if they had a maximum of three titanium plates (12 mm or 19 mm length) with at least 25 mm separation between the fixation plates. To minimise the risk of heating in the presence of titanium plates, scans were performed with a SAR limit set at 90% of normal mode, i.e., a local 10 g SAR limit of 9 W/kg as reported by the scanner with a 1Tx32Rx Nova coil.

These criteria were later updated to allow for two titanium plates and one burr hole cover (18.5 mm diameter) and to permit smaller separations, assessed by an MR safety expert and a neuroradiologist, with scans performed in normal operating mode (scanner-reported 6-min local 10 g SAR limit of 10 W/kg). The total examination duration was limited to one hour, with each individual sequence lasting less than 15 min. The smallest distance between the fixation plates in the participants in this study was 15 mm.

To meet normal mode SAR limits, several sequences required adjustments such as increasing TR, particularly for spectroscopy sequences. The extent to which the sequences required adjustment depended on the location of the region of interest: regions of low B_1_^+^, such as the temporal lobes, were particularly challenging, which meant that some sequences could not be run, even with adjustments.

### 2.6 Neuropathologies

(i) Neuro-oncology, glial brain tumours:
 o Data from three pre-operative patients with different glial tumours with varying molecular profiles and CNS WHO grades.
 o Data from a patient with diagnostic uncertainty post repeat biopsy and craniotomy for a suspected glioma, with glioma subsequently confirmed.
 o Data from two post-operative patients who received post-radical radiotherapy with concomitant and adjuvant temozolomide for glioblastoma.
(ii) Multiple sclerosis:
 o Data from a patient presenting with a tumefactive demyelinating lesion (TDL).
 o Data from a patient presenting with suspected progressive multifocal leukoencephalopathy (PML) after treatment with immune modulators.
(iii) Motor neuron disease:
 o Data from a patient with MND, primary lateral sclerosis (PLS) variant.
(iv) Temporal lobe epilepsy:
 o Data from a patient with temporal lobe epilepsy (TLE).

### 2.7 Histology

The representative histology images provided were obtained from routine diagnostic evaluations using standardised laboratory protocols for fixation and processing of tissue samples, sectioning, and staining protocols for Haematoxylin and Eosin (H&E) staining and immunocytochemistry (ICC), including reagent sources and dilutions, and molecular pathology protocols for fluorescence *in situ* hybridisation (FISH) and isocitrate dehydrogenase (IDH) sequencing.

The spectroscopy voxels tended to be placed over areas of presumed higher-grade tumour, with the voxel sampling the average metabolite ratios within it, with pathology often labelled intra-operatively as to the origin of the sample, and representing a field change/the highest grade within the tumour. The correlation between pathology and surgical site can be challenging, with an experienced neuropathology and neurosurgical team being essential. Voxel placements and scans are usually discussed between neurosurgeons and neuroradiologists, and at multidisciplinary team meetings. We therefore feel confident that the metabolite spectra and concentrations presented reflect the pathology shown.

## 3 Results

7T images and spectra are presented for patients with pre- and post-operative glial brain tumours, along with those with MS, PML, MND, and temporal lobe epilepsy. All sLASER data are presented as raw spectral data, following the post-processing steps detailed above in the methods but prior to fitting. Ratios of the measured metabolites to tCr are presented, alongside the Cramér-Rao Lower Bounds (CRLB) in Table 2 for all the tumour spectra shown and in Table 3 for all the neurodegenerative/neuroinflammatory conditions. The values for SNR and the linewidth of the unsuppressed water peak, in Hertz (Hz), are reported in Table 4 for all the presented spectra.

**Table 2:** Metabolite ratios and CRLBs for tumour spectra.

Metabolite	Pre-op tumour								Post-op tumour		Post-op/post-RT tumour					
	Oligodendroglioma		Astrocytoma - healthy contralateral		Astrocytoma - tumour		Low grade oligo		Astrocytoma		Glioblastoma visit 1		Glioblastoma visit 2		Glioblastoma	
	/Cr+PCr	CRLB (%)	/Cr+PCr	CRLB (%)	/Cr+PCr	CRLB (%)	/Cr+PCr	CRLB (%)	/Cr+PCr	CRLB (%)	/Cr+PCr	CRLB (%)	/Cr+PCr	CRLB (%)	/Cr+PCr	CRLB (%)
tCh	1.27	3	0.20	4	0.37	3	0.24	2	0.22	3	0.27	6	0.25	5	0.28	2
tNAA	0.98	6	1.88	1	1.44	3	1.30	1	0.92	2	0.87	5	1.47	3	1.47	2
Glu			0.76	4	0.52	12	0.71	3	0.51	5	0.64	11	1.02	6	0.89	3
Gln	1.78	6	0.19	18	0.27	26	0.31	8	0.44	7	0.77	10	1.17	6	0.29	9
Ins	1.36	6	0.73	4	0.92	5	0.97	2	1.75	2	0.77	7	0.59	9	0.95	2
Lac	2.63	3			0.85	9	0.08	20	0.25	7	0.36	14	0.25	19	0.31	5
GSH			0.13	12	0.13	25	0.16	7	0.15	9					0.11	10

**Table 3:** Metabolite ratios and CRLBs for neurodegenerative spectra.

Metabolite	MS				MND		TLE	
	TDL		PML					
	/Cr+PCr	CRLB (%)	/Cr+PCr	CRLB (%)	/Cr+PCr	CRLB (%)	/Cr+PCr	CRLB (%)
tCh	0.25	2	0.27	2	0.24	2	0.36	3
tNAA	1.26	1	1.17	1	1.34	1	0.78	4
Glu	1.06	2	0.83	3	0.78	3	0.61	8
Gln	0.44	5	0.53	6	0.37	6		
Ins	0.68	3	1.28	2	1.10	2	0.92	4
Lac	0.24	6	0.65	10	0.14	10		
GSH	0.21	6	0.13	6	0.19	6	0.20	14

**Table 4:** Values of SNR and linewidth for all the acquired spectra.

	Pre-op tumour				Post-op	Post-op/post-RT			MS		MND	TLE
	Oligo	Astro—healthy	Astro—tumour	Low grade	Astro	GBM (1)	GBM (2)	GBM	TDL	PML		
SNR	22	37	14	41	30	10	15	55	42	12	45	6
LW (Hz)	7.8	10.4	6.6	10.4	6.4	6.1	6.8	11.2	12.5	9.6	9.1	10.4

### 3.1 Pre-operative glial brain tumours (three patients)

#### 3.1.1 Patient 1—oligodendroglioma, IDH-mutant, 1p/19q-codeleted (CNS WHO grade 3)

*Presentation:* A female patient in her third decade presented with three to four weeks of high-pressure headaches and bilateral papilloedema.

**Figure 1:**
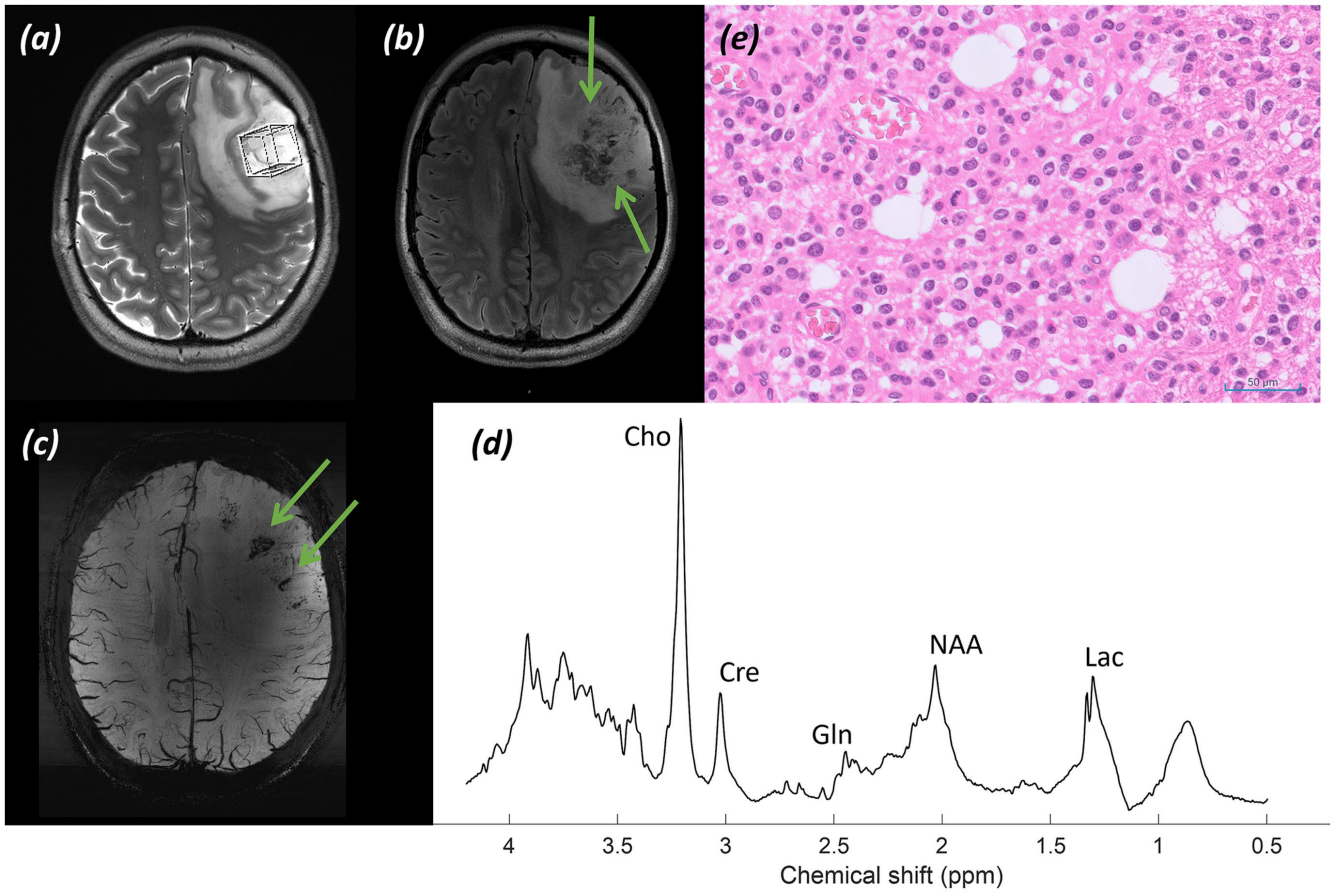
Patient 1: Oligodendroglioma, IDH-mutant, 1p/19q-codeleted (CNS WHO grade 3). **(a)** T_2_-weighted TSE with the MRS voxel marked over the left frontal tumour, **(b)** T_2_-FLAIR (green arrows indicate heterogeneous tumour), **(c)** SWI (green arrows indicate susceptibility artefact from intra-tumoral calcification), **(d)** sLASER spectrum, **(e)** Histology (H&E; ×20).

*Imaging:* MRI demonstrated a heterogeneous lesion with calcification and marked vascularity involving the cortex and subcortical white matter of the left frontal lobe, centred on the left middle and inferior frontal gyri and extending inferiorly to the anterior insula, engulfing the expected location of Broca’s area. Structural imaging raised suspicion of oligodendroglioma. Images shown in Figure 1 include (a) T_2_-weighted TSE with the MRS voxel marked, (b) T_2_-FLAIR, and (c) SWI.

*MRS findings:* The sLASER spectrum from the lesion (Figure 1d) demonstrated a reversal of the normal tNAA/tCho and tCho/tCr ratios, with a decrease in NAA, a lactate peak, no rise in Ins, and a decrease in Cr. An increase in the Gln peak was also noted. These appearances supported a higher-grade glial tumour and, together with the structural imaging features, favoured oligodendroglioma. In addition, a 2HG peak was detected, suggesting the presence of an IDH-mutant glioma; however, the acquisition was not optimised for this, with a short TE resulting in a high fitting error (CRLB 55%).

*Histology:* Figure 1e (H&E; ×20) shows small to medium-sized cells with rounded nuclei, occasionally cleared cytoplasm, and scattered mitotic figures.

*Final diagnosis and integrated pathology:* Histopathology from the stereotactic biopsy confirmed oligodendroglioma, IDH-mutant and 1p/19q-codeleted (CNS WHO grade 3; MGMT promoter methylated).

#### 3.1.2 Patient 2—astrocytoma, IDH-mutant (CNS WHO grade 3)

*Presentation:* A male patient in his fifth decade presented with a tonic–clonic seizure.

**Figure 2:**
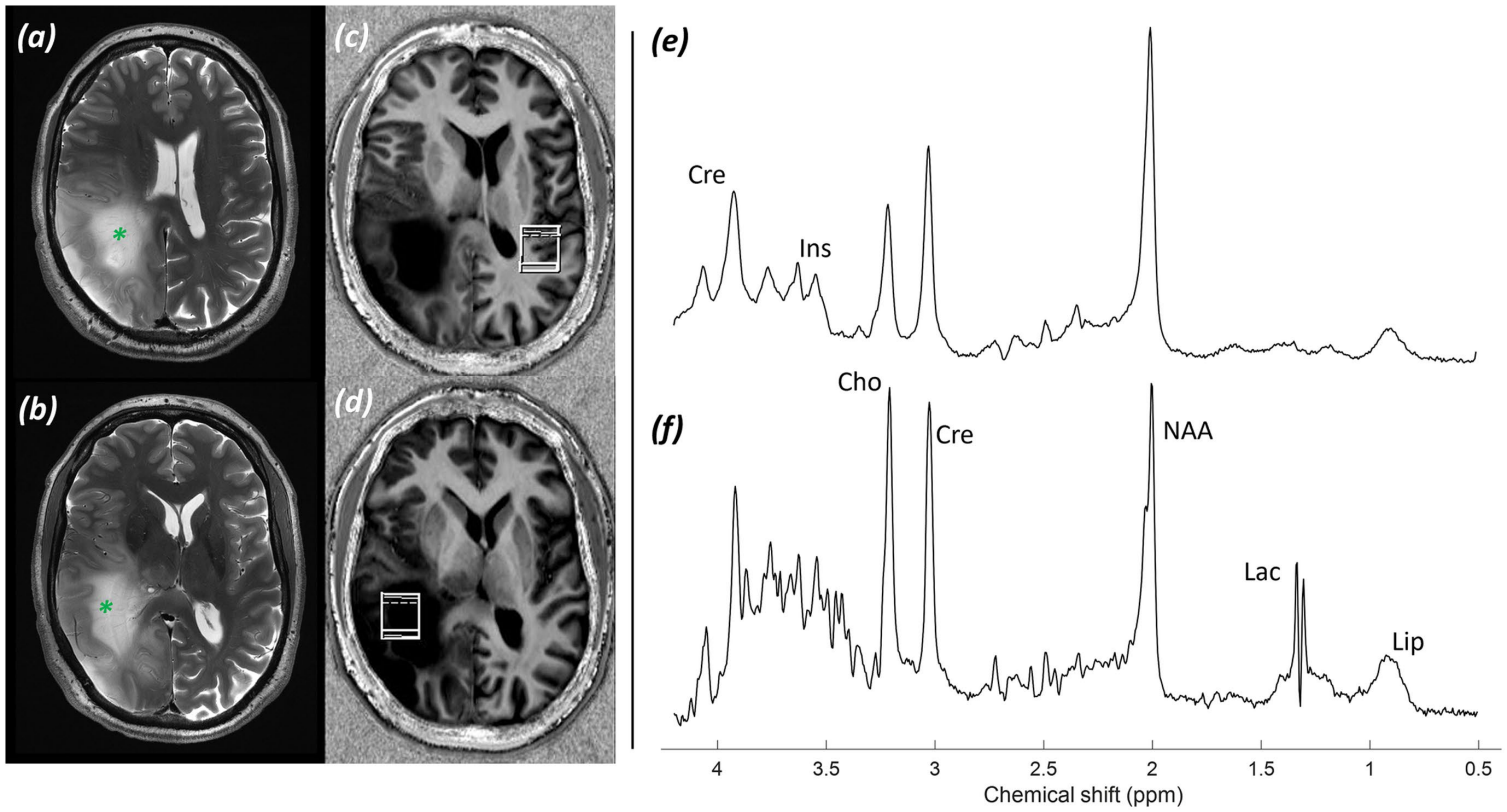
Patient 2: Astrocytoma, IDH-mutant (CNS WHO grade 3). **(a,b)** T_2_-weighted TSE images at two levels (green asterisk* marks tumour), **(c)** MP2RAGE UNI with control voxel over contralateral normal white and grey matter, **(d)** MP2RAGE UNI with tumour voxel over the right peritrigonal lesion, **(e)** sLASER spectrum from the control voxel, **(f)** sLASER spectrum from the lesional voxel.

*Imaging:* MRI demonstrated a heterogeneous right peritrigonal space-occupying lesion with some preserved brain matrix and vasculature and ill-defined infiltrative margins. Images shown in Figure 2 include (a, b) T_2_-weighted TSE images and (c, d) MP2RAGE images demonstrating the position of the non-lesional control voxel (c) and the lesional voxel (d).

*MRS findings:* The non-lesional voxel showed an sLASER spectrum with normal metabolite concentrations (Figure 2e). The lesional voxel (Figure 2f) demonstrated decreased tNAA and increased tCho peaks, along with lactate and lipid peaks. Ins and Gln peaks were low. In addition, a 2HG peak was detected, suggesting the presence of an IDH-mutant glioma; however, the acquisition was not optimised for this, with a short TE, resulting in a high fitting error (CRLB 33%). These findings supported a higher*-*grade astrocytoma.

**Figure 3:**
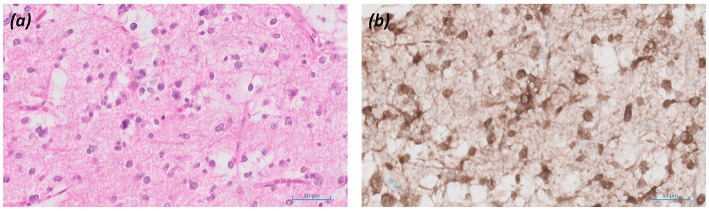
Patient 2: Astrocytoma, IDH-mutant (CNS WHO grade 3). **(a)** Histology (H&E; ×20), **(b)** Immunostaining for mutant form of IDH-1 protein (×20).

*Histology and immunostaining:* Figure 3a revealed a diffusely infiltrating tumour composed of elongated, spindle*-*shaped cells with occasional mitotic figures present (H&E; ×20). Figure 3b showed immunostaining results for the mutant form of isocitrate dehydrogenase-1 (IDH-1) protein (×20).

*Final diagnosis and integrated pathology:* Histopathology confirmed astrocytoma, IDH-mutant (CNS WHO grade 3; MGMT promoter methylated).

#### 3.1.3 Patient 3—oligodendroglioma, IDH-mutant and 1p/19q-codeleted (CNS WHO grade 2)

*Presentation:* A female patient in her early third decade was investigated for exacerbation of migraines.

**Figure 4:**
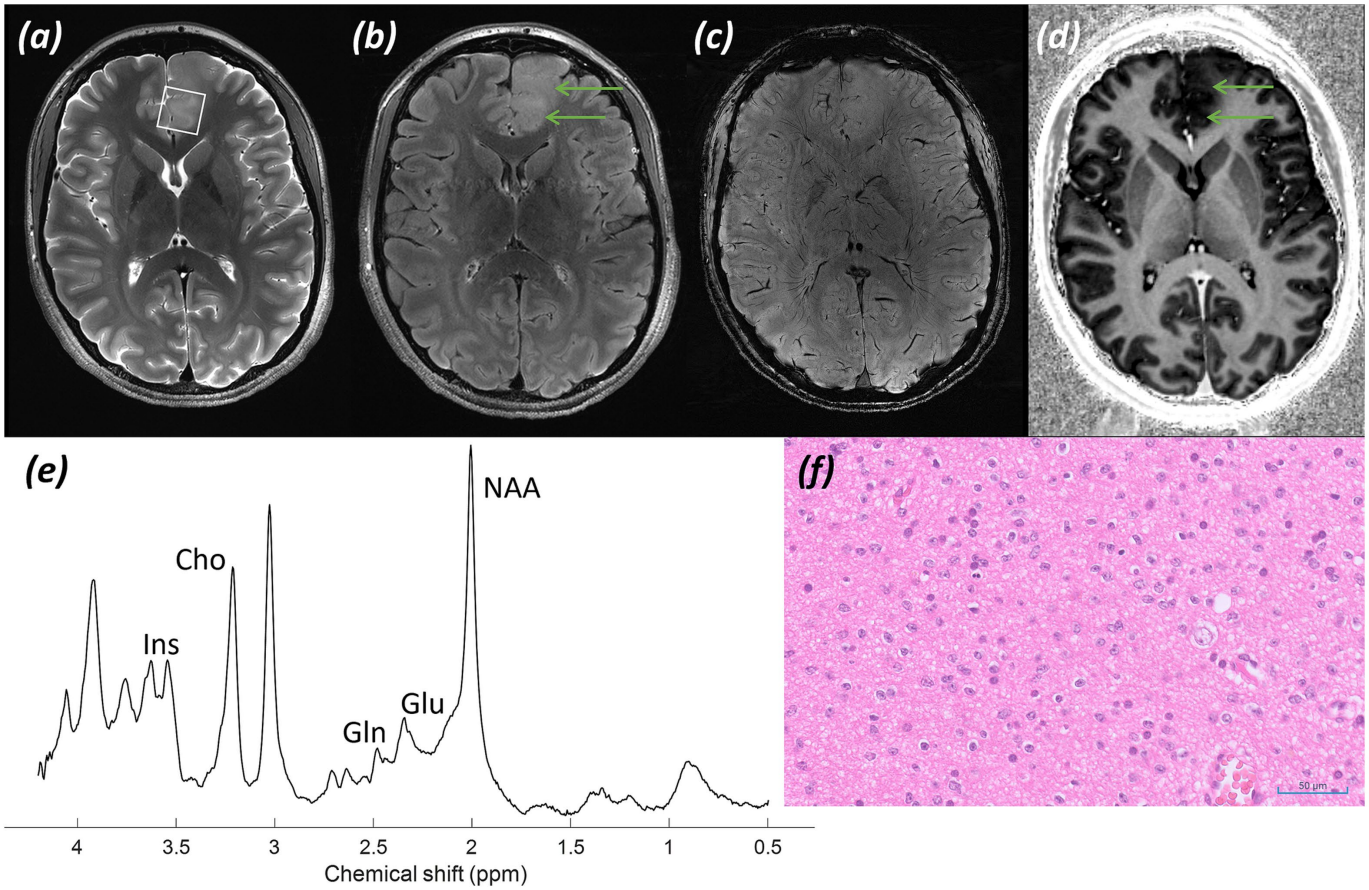
Patient 3: Oligodendroglioma, IDH-mutant, 1p/19q-codeleted (CNS WHO grade 2). **(a)** T_2_-weighted TSE image, MRS voxel location marked over the left anterior cingulate space-occupying lesion, **(b)** T_2_*-*FLAIR image (green arrows delineate tumor), **(c)** SWI image, **(d)** T_1_-weighted MP2RAGE UNI image (green arrows delineate tumor), **(e)** sLASER spectrum, **(f)** Histology (H&E; ×20).

*Imaging:* Structural sequences showed a relatively well-defined homogeneous left anterior cingulate space-occupying lesion involving cortex and subcortical white matter with subtle expansion, with no post-contrast enhancement and no susceptibility artefact indicating calcification or blood products. Images shown in Figure 4 include (a) a T_2_-weighted TSE with the MRS voxel marked, (b) a T_2_-FLAIR, (c) an SWI image, and (d) a T_1_-weighted MP2RAGE UNI post gadolinium-based contrast image.

*MRS findings:* The sLASER spectrum (Figure 4E) showed only a minor decrease in tNAA and an increase in tCho, along with a raised Ins peak, with no high-grade features. A raised Gln peak was also noted. In addition, a small 2HG peak was detected, suggesting the presence of an IDH-mutant glioma; however, the acquisition was not optimised for this, with a short TE, resulting in a high fitting error (CRLB 89%). These findings on MRS supported a lower-grade glial tumour.

*Histology:* Figure 4F shows a population of tumour cells with typically rounded nuclei and occasional cytoplasmic clearing (H&E; ×20).

*Final diagnosis and integrated pathology*: Histopathology confirmed an oligodendroglioma, IDH-mutant and 1p/19q-codeleted (CNS WHO grade 2; MGMT promoter methylated).

### 3.2 Post-operative, pre-treatment glial brain tumour (one patient)

#### 3.2.1 Patient 4—glioblastoma, IDH-wildtype (CNS WHO grade 4)

*Presentation:* A female patient in her sixth decade initially presented 6 years earlier with ataxia, nystagmus, and light-headedness. Repeated biopsies and craniotomies had failed to yield a definitive histological diagnosis. Titanium cranioplasty fixation plates were present.

**Figure 5:**
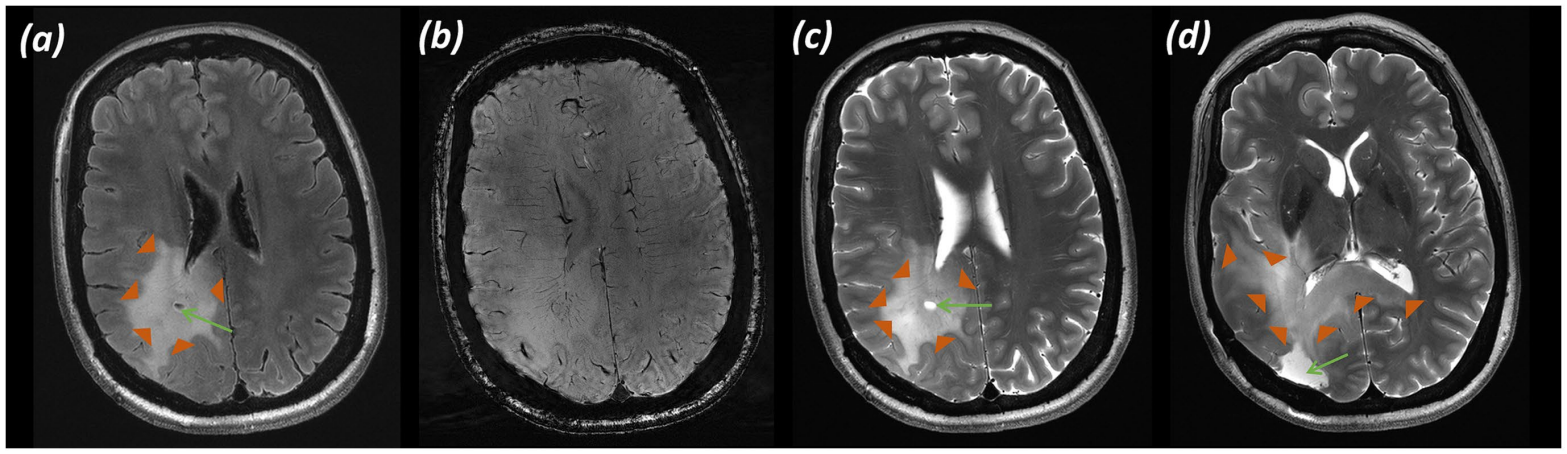
Patient 4: Glioblastoma, IDH-wildtype (CNS WHO grade 4). **(a)** T_2_*-*FLAIR image, **(b)** SWI image, and **(c,d)** T_2_-weighted TSE images at different slice levels through tumor and biopsy cavities. Orange arrowheads delineate tumor; green arrows mark resection cavities.

**Figure 6:**
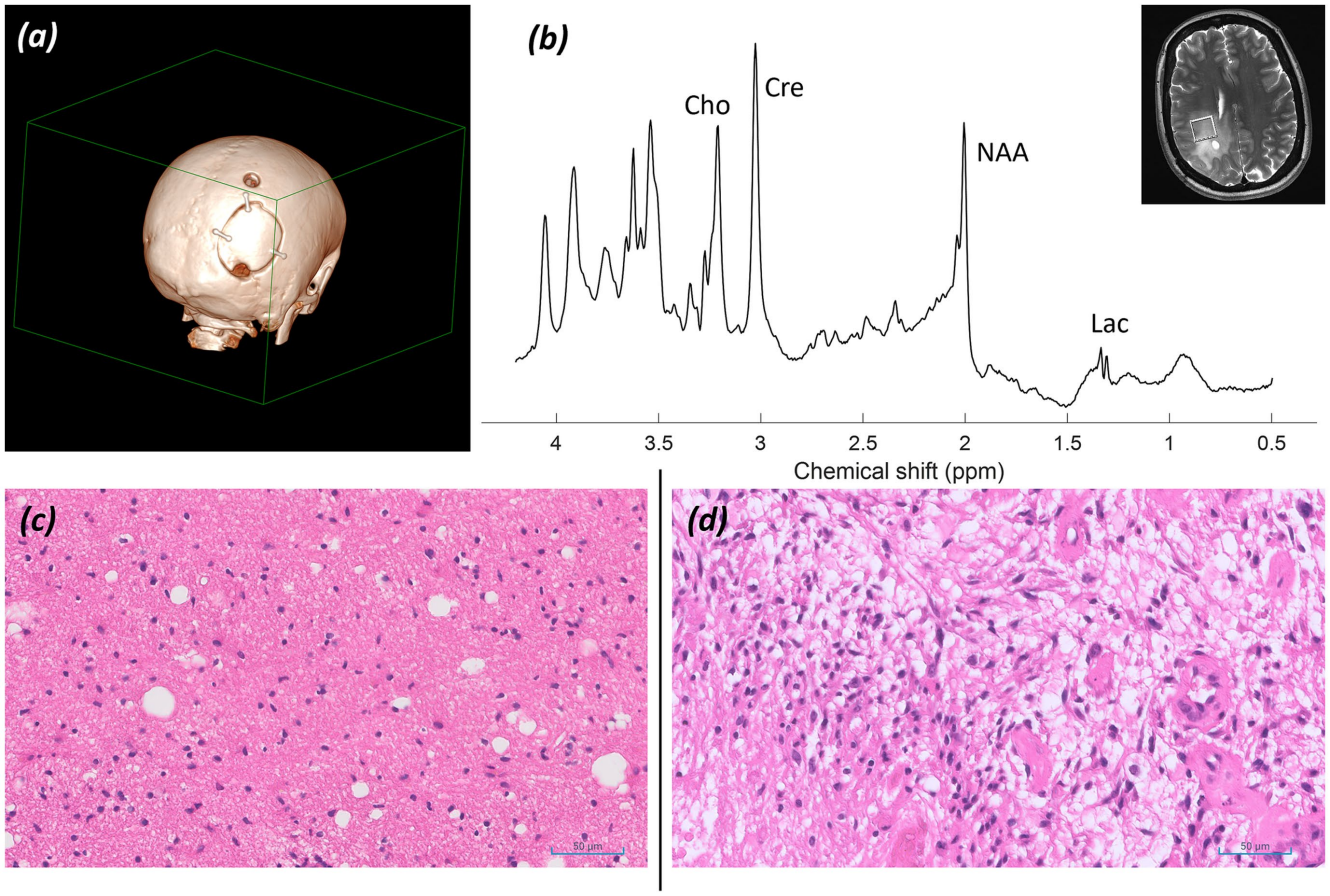
Patient 4: Glioblastoma, IDH-wildtype (CNS WHO grade 4). Further imaging and histology. **(a)** 3D CT reconstruction of the skull showing the craniotomy with the position of the metal skull fixation plates, **(b)** sLASER spectrum, with the voxel position inset. Histology at first presentation **(c)** (×20; H&E), and at later debulking surgery **(d)** (×20; H&E).

*Imaging:* Structural MRI showed extensive abnormal white matter signal centred on the right posterior parietal, occipital, and posterior temporal lobes surrounding the trigone and posterior horn of the right lateral ventricle, extending anteriorly to the posterior insula, posterior limb of the internal and external capsule, and thalamus. Foci of cerebromalacia, gliosis, and haemosiderin staining relating to previous surgical interventions and biopsies were noted. Due to previous surgical intervention and associated skull fixation plates, imaging was performed at 90% of the basic SAR level. Images are shown in Figures 5, 6.

*MRS findings:* sLASER MRS was performed from multiple lesional voxels, with a representative spectrum shown in Figure 6. The spectrum showed early reversal of the normal tNAA/tCho and tCho/tCr ratios and a small lactate peak, supporting malignancy. No 2HG peak was detected, suggesting an IDH-wildtype tumour.

*Histology:* Histology at first presentation (Figure 6c) shows an increase in cellularity in the white matter (×20; H&E); and at later debulking surgery (Figure 6d) shows a cellular tumour composed of atypical astroglial forms with frequent mitotic figures and foci of necrosis (×20; H&E).

*Final diagnosis and integrated pathology:* Corresponding histology sections from the initial biopsy and later debulking surgery illustrate the evolution from non-diagnostic findings (Figure 6c) to a tumour subsequently classified as glioblastoma, IDH-wildtype (CNS WHO grade 4; MGMT promoter unmethylated) (Figure 6d).

### 3.3 Post-operative and post-radiotherapy glioblastoma (two patients)

#### 3.3.1 Patient 5—glioblastoma, IDH-wildtype (CNS WHO grade 4)

*Presentation:* A male patient in his sixth decade was investigated following a right temporal craniotomy and radiotherapy for neurosurgical debulking of glioblastoma, followed by radical chemoradiation (60 Gy in 30 fractions with concomitant temozolomide) and six cycles of adjuvant temozolomide.

**Figure 7:**
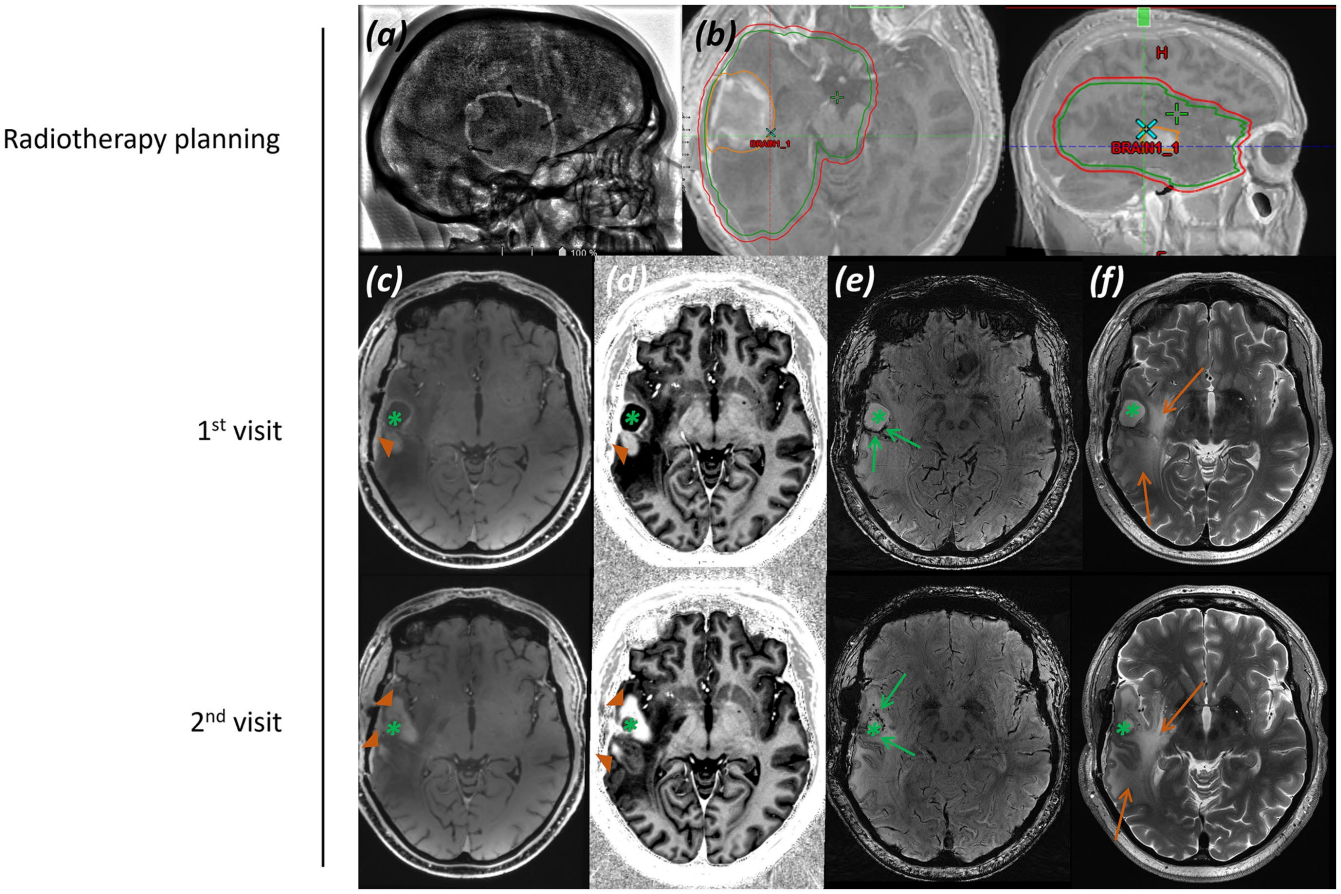
Patient 5: Glioblastoma, IDH-wildtype (CNS WHO grade 4), post-operative and post-radiotherapy. **(a)** CT image displaying craniotomy and titanium fixation plates, **(b)** radiotherapy planning maps in axial and sagittal planes, with gross tumor volume (GTV, orange), clinical target volume (CTV, green), and planning target volume (PTV, red) marked, and **(c)** second inversion time image from a T_1_-weighted MP2RAGE acquisition post-gadolinium contrast, **(d)** T_1_-weighted MP2RAGE UNI image post-gadolinium contrast, **(e)** SWI, and **(f)** T_2_-weighted TSE image, all at two time points 5 months apart, one month and six months post completion of radiotherapy. (Green asterisk* indicates resection cavity; green arrow indicates blood degradation products surrounding the resection cavity; orange arrowhead indicates post-contrast enhancement; orange arrow indicates abnormal white matter).

*Imaging:* Imaging presented was performed one month and seven months after completion of radiotherapy. Post-operative imaging demonstrated a resection cavity centred upon the right superior and middle temporal gyrus, with surrounding post-surgical haemosiderin staining and non-enhancing abnormal T_2_-weighted hyperintensity in the temporal lobe. Some adjacent enhancement was evident. At follow-up five months later, the suspected residual or recurrent tumour had increased in size, with thick nodular peripheral enhancement extending to the right Sylvian fissure and insula, along with increased non-enhancing white matter signal abnormality. Imaging is shown in Figure 7.

**Figure 8:**
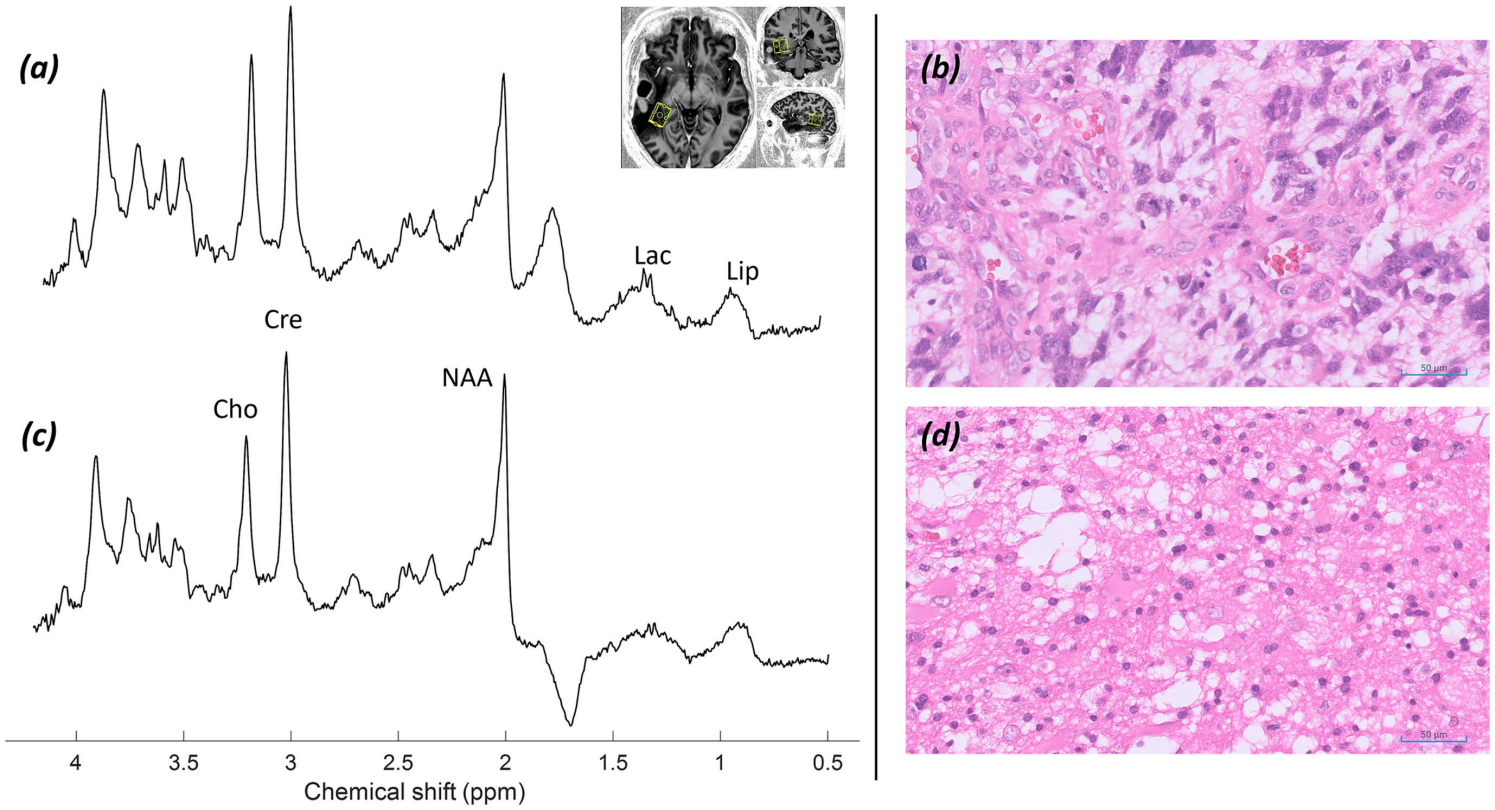
Patient 5: Glioblastoma, IDH-wildtype (CNS WHO grade 4), post-operative and post-radiotherapy. Further MRS and histology. **(a)** sLASER spectrum acquired during the patient’s first visit, with the voxel position marked in three planes of an MP2RAGE UNI image. **(b)** Histology from the initial surgery showing a highly cellular, poorly differentiated tumour composed of highly pleomorphic, atypical astroglial cells with frequent mitotic figures and florid vascular proliferation, typical of a glioblastoma (H&E; ×20). **(c)** sLASER spectrum from the second visit five months later (lesional voxel acquired in the same position as at visit 1). **(d)** Histology following repeated surgery (H&E; ×20).

*MRS findings:* sLASER MRS from a voxel placed adjacent to the resection cavity over abnormal but non-enhancing white matter within the radiation field demonstrated alterations in the tNAA/tCho and tCho/tCr ratios, in addition to lactate and lipid peaks (Figure 8a). These appearances raised concern for tumour progression or recurrence, with a possible element of radiation necrosis.

*Histology:* Histology from the initial surgery (Figure 8b) shows a highly cellular, poorly differentiated tumour composed of highly pleomorphic, atypical astroglial cells with frequent mitotic figures and florid vascular proliferation, typical of a glioblastoma (H&E; ×20). Histology following repeated surgery (Figure 8d) shows a tumour of lower cellularity, without the high-grade proliferative activity.

*Final diagnosis and integrated pathology:* Histology from the original debulking surgery confirmed a glioblastoma, IDH-wildtype (CNS WHO grade 4, MGMT promoter methylated). Subsequent surgery for suspected recurrence showed a lesion without higher*-*grade features (Figure 8d).

#### 3.3.2 Patient 6—glioblastoma, IDH-wildtype (CNS WHO grade 4)

*Presentation:* A male patient in his fourth decade was investigated following a left parietal craniotomy for tumour debulking, followed by radical chemoradiation (60 Gy in 30 fractions with concomitant temozolomide) and six cycles of adjuvant temozolomide.

*Imaging:* Imaging presented was performed one and seven months after completion of radiotherapy.

**Figure 9:**
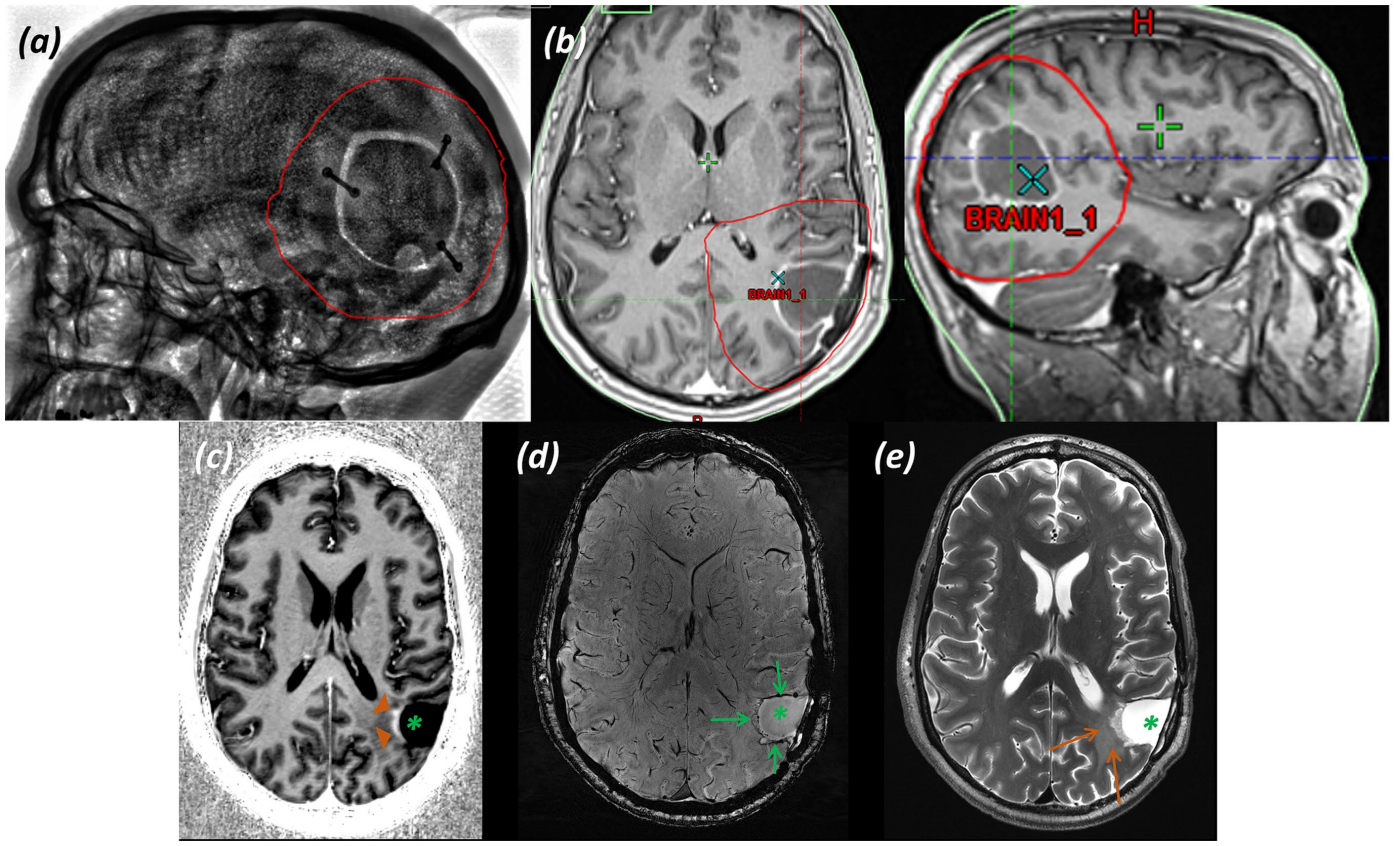
Patient 6: Glioblastoma, IDH-wildtype (CNS WHO grade 4), post-operative and post-radiotherapy. **(a)** CT image displaying craniotomy metalware, **(b)** Radiotherapy planning maps in axial and sagittal planes, with PTV (red) marked, and **(c)** T_1_-weighted MP2RAGE UNI image post-gadolinium contrast, **(d)** SWI, and **(e)** T_2_-weighted TSE image through the level of the resection cavity. (Green asterisk* indicates resection cavity; green arrow indicates blood degradation products surrounding the resection cavity; orange arrowhead indicates post-contrast enhancement; orange arrow indicates abnormal white matter).

Post-operative imaging demonstrated a resection cavity centred on the left parietal lobe, with adjacent enhancement and abnormal white matter signal within the radiotherapy field. Imaging is shown in Figure 9.

**Figure 10:**
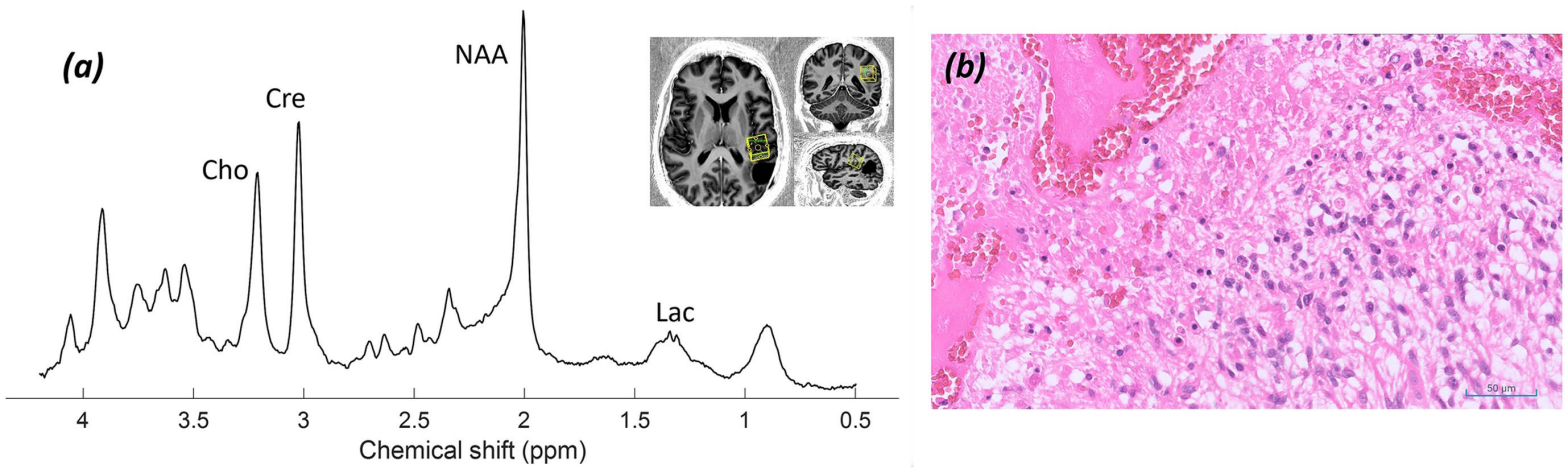
Patient 6: glioblastoma, IDH-wildtype (CNS WHO grade 4), post-operative and post-radiotherapy. Further MRS and histology. **(a)** sLASER spectrum, with voxel position marked in axial, coronal, and sagittal planes of an MP2RAGE UNI post-gadolinium contrast image. **(b)** Histology from the original surgery preceding radiotherapy (H&E; ×20).

*MRS findings:* sLASER MRS from a voxel placed over non-enhancing abnormal white matter adjacent to the resection cavity demonstrated no significant alterations in the tNAA/tCho and tCho/tCr ratios, with only a small lactate peak (Figure 10A). These appearances were felt likely to reflect post-radiotherapy effects and radiation necrosis.

*Histology:* Histology from the original surgery preceding radiotherapy (Figure 10b) shows a largely necrotic, poorly differentiated tumour with frequent mitotic figures (H&E; ×20).

*Integrated pathology*: Histology from the original surgery (Figure 10b) confirmed glioblastoma, IDH-wildtype (CNS WHO grade 4; MGMT promoter methylated).

*Clinical outcome and final diagnosis:* The patient remains recurrence-free nearly two years after completion of radiotherapy. Structural imaging and MRS post-radiotherapy reflected radiation necrosis.

### 3.4 Multiple sclerosis (two patients)

#### 3.4.1 Patient 7—tumefactive demyelinating lesion

*Presentation:* A female patient in her third decade presented with a large solitary lesion in the right centrum semiovale of uncertain aetiology, following two subsequently inconclusive biopsies.

**Figure 11:**
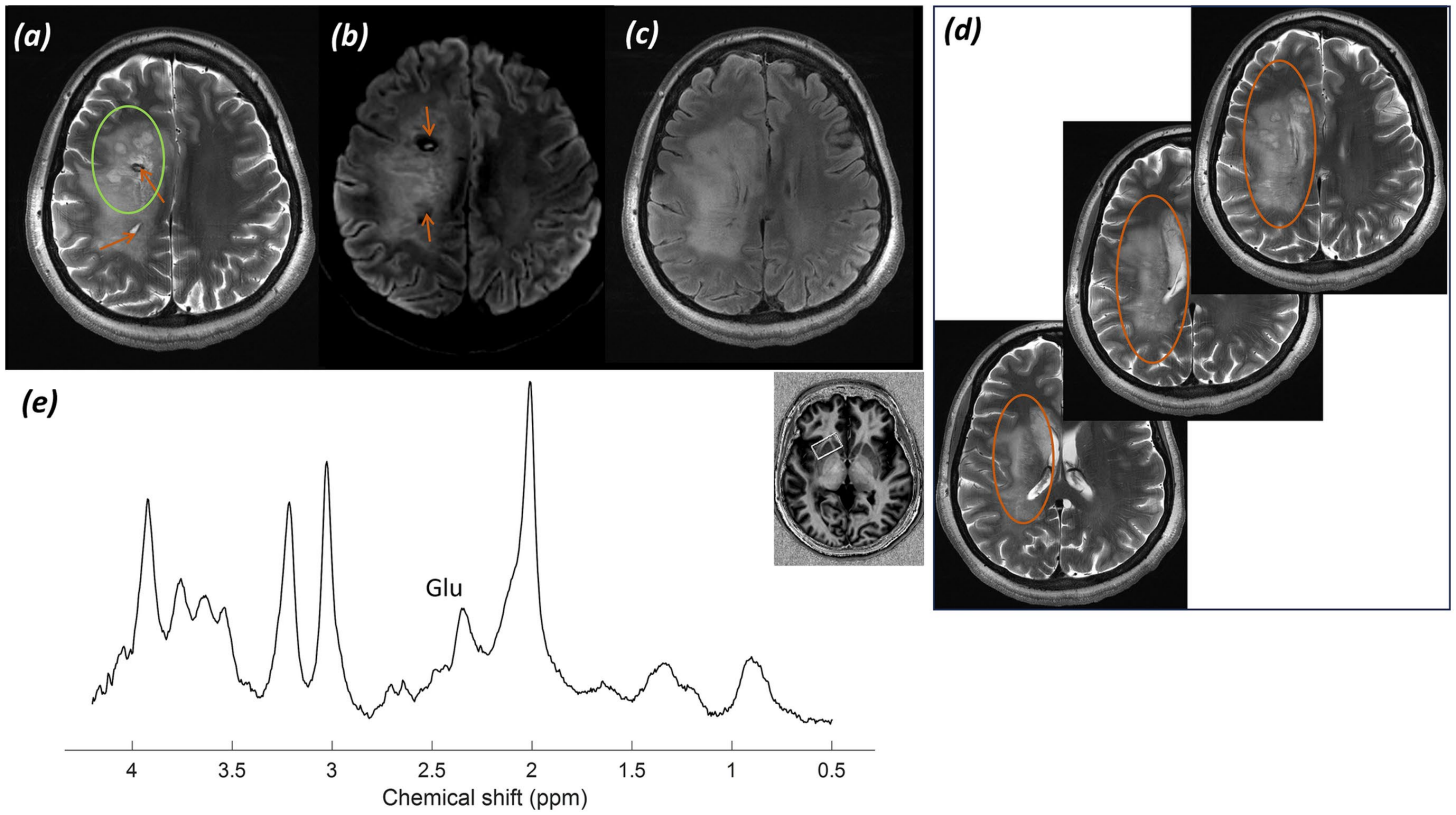
Patient 7: Tumefactive demyelinating lesion (multiple sclerosis). **(a)** T_2_-weighted TSE, **(b)** RESOLVE-DWI, **(c)** T_2_ FLAIR image from the heterogeneous component of the white matter lesion (green circle delineates this heterogeneous area, with orange arrows pointing to biopsy cavities and haemosiderin staining), **(d)** T_2_-weighted imaging at three levels, demonstrating the extent of the TDL (orange arrows), **(e)** sLASER spectrum avoiding areas of post-biopsy haemosiderin deposition at the antero-caudal part of the TDL (a version of this figure appeared in Fullerton et al., 2024), with the voxel location inset.

*Imaging:* MRI showed a heterogeneous lesion with areas of raised diffusion-weighted signal extending to juxtacortical white matter, with a biopsy tract and blood products in the antero-superior part of the lesion. Images shown in Figure 11 include (a) a T_2_-weighted TSE, (b) a RESOLVE-DWI image, (c) a T_2_-FLAIR image, and (d) three T_2_-weighted TSE images at different levels of the lesion.

*MRS findings:* Single voxel sLASER MRS was acquired with the voxel placed over the antero-caudal component of the right centrum semiovale lesion extending to corona radiata white matter and deep grey nuclei, avoiding areas of post-biopsy haemosiderin deposition (Figure 11e). The spectrum demonstrated a glutamate peak, which was not visible at 3T MRS.

*Final diagnosis:* These findings supported a diagnosis of tumefactive demyelinating lesion, with subsequent development of new characteristic demyelinating plaques further supporting a diagnosis of multiple sclerosis.

#### 3.4.2 Patient 8—progressive multifocal leukoencephalopathy (PML)

*Presentation:* A female patient in her fourth decade with known MS, treated with natalizumab, who became John Cunningham Virus (JCV) positive.

**Figure 12:**
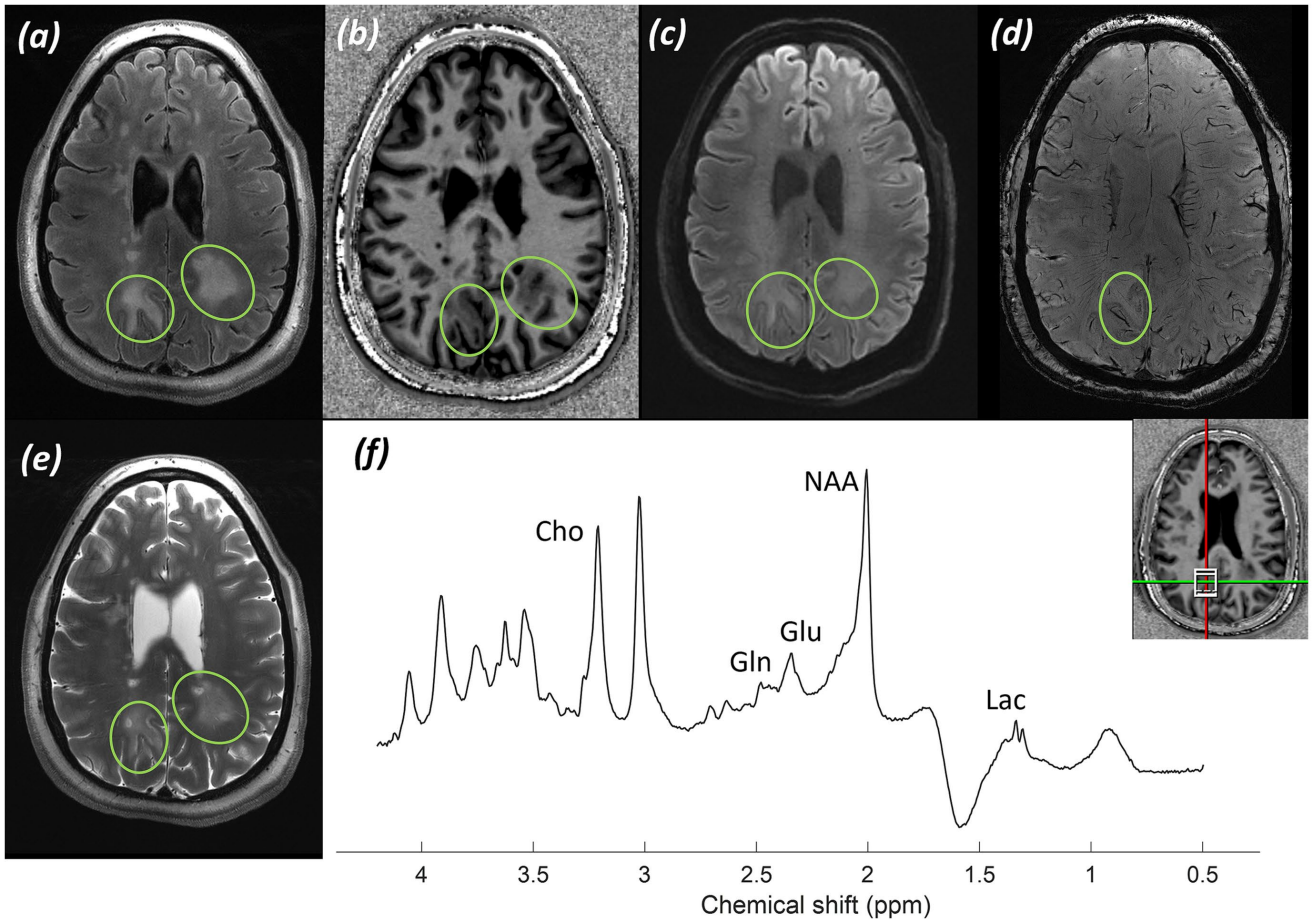
Patient 8: Progressive multifocal leukoencephalopathy (multiple sclerosis). **(a)** T_2_ FLAIR, **(b)** T_1_-weighted MP2RAGE UNI image, **(c)** RESOLVE-DWI, **(d)** SWI, **(e)** T_2_-weighted TSE, and **(f)** sLASER spectrum with the voxel location corresponding to the spectrum displayed marked on an MP2RAGE UNI image. (A version of the structural images shown appeared in Fullerton et al., 2024). Areas suggestive of PML in the right and left hemispheres are shown with green circles.

*Imaging:* MRI showed areas of juxtacortical signal abnormality, ill-defined towards white matter, centred on the medial occipital and parietal lobes, with a punctate-like “milky-way” appearance adjacent to the cortex. Images shown in Figure 12 include (a) a T_2_-FLAIR image, (b) an MP2RAGE UNI image, (c) a RESOLVE-DWI image, (d) an SWI image, and (e) a T_2_-weighted TSE.

*MRS findings:* An sLASER spectrum (Figure 12f) was obtained from a voxel placed over the extensive signal abnormality at the level of the medial right and left occipital lobe, as shown in an inset axial MP2RAGE UNI image. The spectrum demonstrates a lactate peak. A raised Gln peak, with some decrease in tNAA and increase in tCho peaks, was also noted.

*Final diagnosis:* A diagnosis of PML was made.

### 3.5 Motor neuron disease (one patient)

#### 3.5.1 Patient 9—motor neuron disease (MND), primary lateral sclerosis (PLS) variant

*Presentation:* A male in his seventh decade who was diagnosed 6 years prior to imaging with the PLS variant of MND.

**Figure 13:**
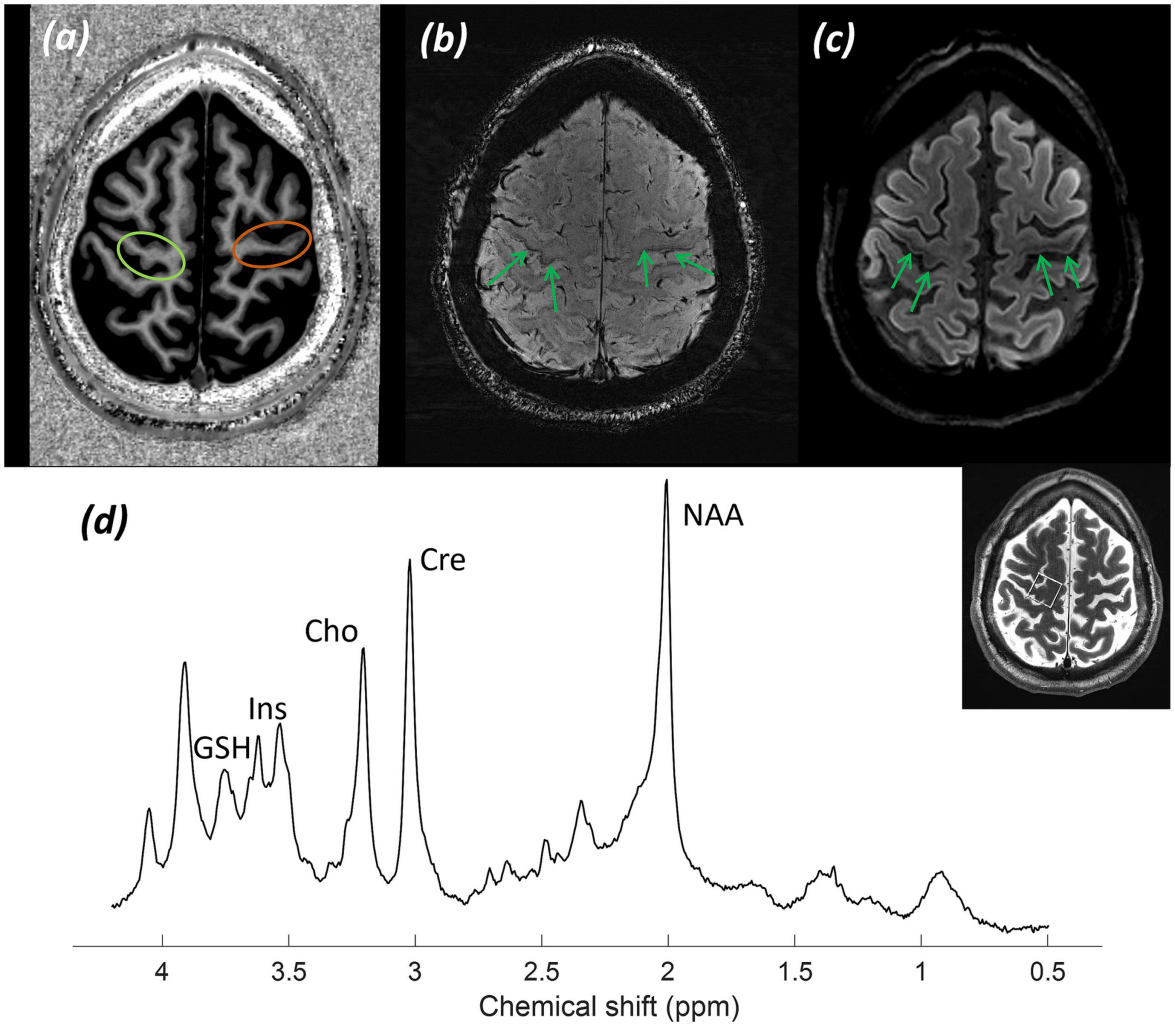
Patient 9: Motor neuron disease (primary lateral sclerosis variant). **(a)** T_1_-weighted MP2RAGE UNI image with atrophic left (green circle) and right (orange circle) hand and foot motor cortex, **(b)** SWI, showing a “motor band sign” (green arrows), and **(c)** RESOLVE-DWI, with altered diffusion-weighted signal reflecting mineral deposition in the right and left hemispheric motor cortex (green arrows), and **(d)** A sLASER spectrum from the left hand motor cortex, with the voxel location marked on a T_2_-weighted TSE image.

*Imaging:* Figure 13 shows (a) an MP2RAGE UNI image demonstrating significant atrophy of the motor cortex, particularly the barely identifiable, markedly atrophic right-hemispheric hand motor cortex, corresponding to the more pronounced left-sided upper limb symptoms experienced; (b) an SWI, where a rim of susceptibility artefact can be seen extending along the right and left hemispheric motor cortex, a so-called “motor band sign” secondary to mineral deposition from neurodegeneration; and (c) a RESOLVE-DWI showing a noticeable decrease in diffusion-weighted signal and hypointensity affecting the right and left hemispheric motor cortex, reflecting mineral deposition at this level.

*MRS findings:* Figure 13d shows an sLASER spectrum from a voxel positioned over the motor cortex, as indicated in an inset axial TSE image. The spectrum shows a decreased concentration of tNAA and an associated decreased tNAA/tCr ratio. Additionally, a raised inositol (Ins) peak and glutathione (GSH) are observed. These appearances on MRS are consistent with MND.

*Final diagnosis:* Findings consistent with MND, PLS variant.

### 3.6 Temporal lobe epilepsy (one patient)

#### 3.6.1 Patient 10—temporal lobe epilepsy with amygdala enlargement

*Presentation:* A female in her third decade who presented with new-onset epilepsy accompanied by emotional changes.

**Figure 14:**
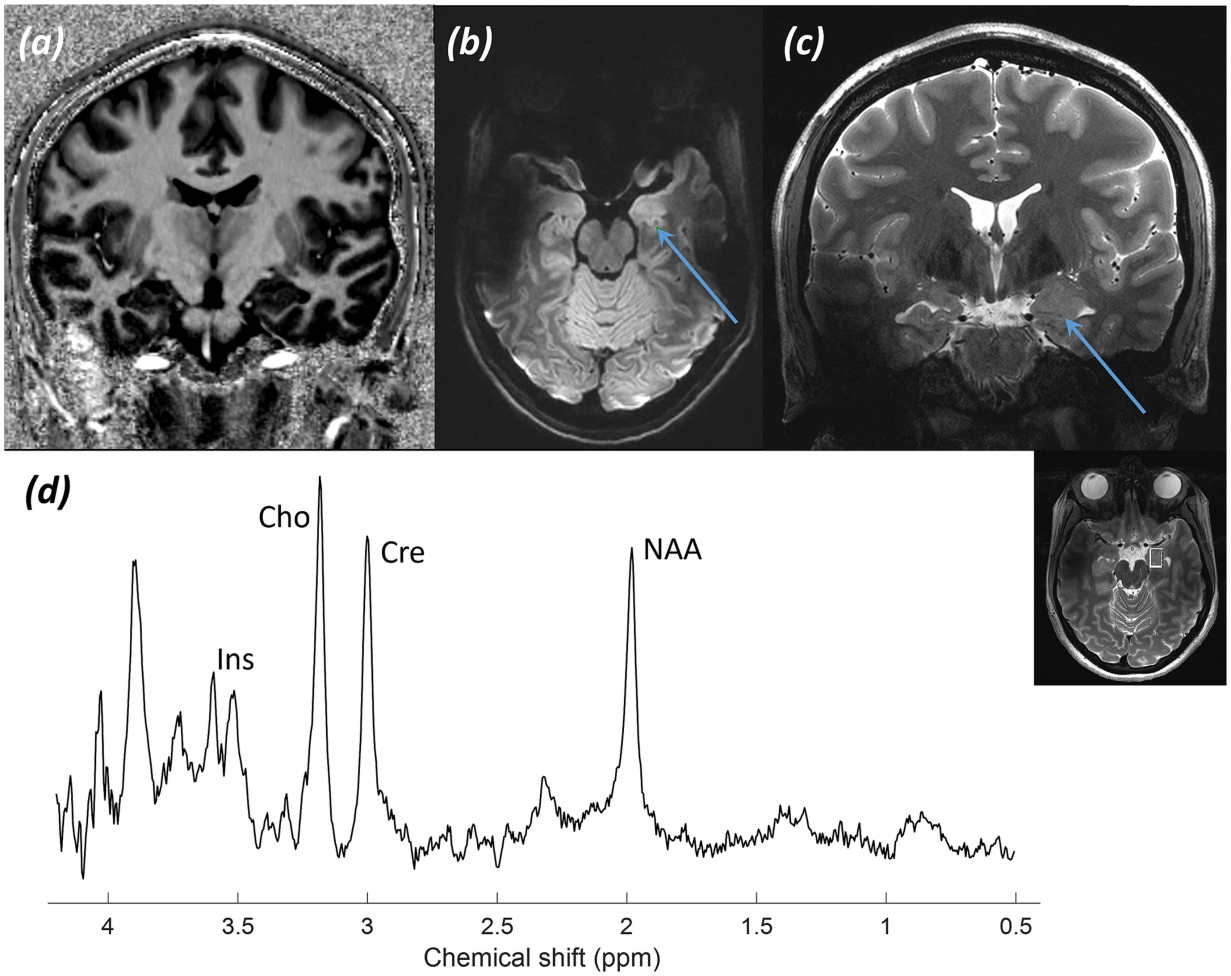
Patient 10: Temporal lobe epilepsy with amygdala enlargement. **(a)** T_1_-weighted MP2RAGE UNI image of the hippocampi, **(b)** Transverse T_2_-FLAIR image at the level of the amygdala (blue arrow) and hippocampi, and **(c)** Coronal T_2_-TSE image, where the blue arrows point to subtle swelling of the left amygdala, and **(d)** sLASER spectrum with the voxel placed over the left amygdala, with the voxel location marked on a T_2_-TSE image.

*Imaging:* Figure 14 shows (a) coronal MP2RAGE UNI, (b) axial T_2_-FLAIR, and (c) coronal T_2_-TSE demonstrating swelling of the left amygdala on T_2_-weighted FLAIR and TSE images.

*MRS findings:* Figure 14d shows the sLASER acquisition. This was technically challenging due to the location within the anterior inferior temporal lobe and skull base, as shown in an inset axial TSE image. After appropriate shimming, a spectrum was obtained demonstrating a decrease in the tNAA peak when compared to choline (tCho) and creatine (tCr), along with some increase in inositol.

*Final diagnosis:* Appearances suggest temporal lobe epilepsy with amygdala enlargement, with a differential diagnosis of a hamartomatous or low*-*grade lesion.

## 4 Discussion

7T imaging and sLASER MRS were performed in patients presenting with brain tumours (including pre- and post-surgery and post-treatment), demyelinating diseases (tumefactive MS and PML), MND, and epilepsy.

The acquired 7T ^1^H spectra were of good quality, with high SNR, low linewidths, and greater spectral resolution than is achievable with lower field strength MRS, offering useful neurochemical information in the presented pathologies. Good spectra could be achieved even close to the skull base in the temporal regions after appropriate optimisation, where B_1_ and B_0_ field inhomogeneities and poorer SNR affect image quality. Good spectra could also be obtained in patients with metalware, requiring adaptation of imaging parameters to adjust to SAR restrictions. MRS at 7T has a complex workflow requiring optimisation tailored to anatomical regions, safety conditions, RF coils, and clinical questions. It is a technique that requires a level of familiarity from operators to avoid inaccurate results. High-quality spectra, making use of the opportunities 7T has to offer, are crucial for increasing clinical applications. The examples provided illustrate the capabilities of MRS at UHF.

Figures 1–4 show three somewhat different cases of glial tumours. The tumour spectrum in Figure 1 displays the characteristic tNAA/tCho reversal, as well as a build-up of lactate in the tumour tissue due to increased anaerobic metabolism. In some higher-grade gliomas with areas of heterogeneity and radiological features of cystic degeneration, there is also an increase in Gln evident, as previously described (Hangel et al., 2019), and this is also seen in Figure 1. An increase in Gln is difficult to appreciate at lower clinical field strengths due to the overlap of the Gln peak with Glu, owing to insufficient spectral resolution at 1.5 T and 3T. The value of the Glx peak measured at these field strengths (a combination of signal from the overlapping Gln and Glu) in tumours has been observed to show no change relative to normal-appearing white matter (Li et al., 2015). In addition, a noticeable decrease in the Cr peak is seen. The Cr peak, composed of creatine and phosphocreatine, reflects metabolism. It has been speculated that a decrease in Cr reflects energy metabolism and tumour aggressiveness, with Cr decreased in higher-grade glial tumours due to tissue hypoxia, metabolic changes, and compromised vascular integrity; frequently seen in conjunction with an increased Cho peak, as here, reflecting membrane turnover, and a lactate peak, reflecting anaerobic glycolysis (Cai et al., 2017; Lupo et al., 2007). The spectrum is that of a suspected high-grade lesion, which, in conjunction with structural appearances (McCarthy et al., 2022a) and detection of 2HG (Emir et al., 2016), would be consistent with a high-grade oligodendroglioma, CNS WHO grade 3, which was confirmed on histology.

Appearances based on structural imaging and MRS, presented in Figure 2, show high-grade features with alterations in tNAA/tCho and tNAA/tCr ratios, in addition to lactate and lipid peaks, though with no rise in Gln and some presence of 2HG. In contrast, these findings are consistent with the subsequent pathology of a Grade 3 IDH-mutant astrocytoma.

Minor alterations in the lesional spectrum were noted in Figure 4, with no features of a higher grade lesion, in contrast to Figures 1, 2. In particular, there was no significant rise in Cho and decrease in NAA peak, no reversal of the tNAA/tCho ratio, and no notable lactate or lipid peak. Ins was raised, a finding associated with lower grade lesions, along with a raised Gln peak, supporting an oligodendroglioma. Again, some 2HG could be seen, in keeping with an IDH-mutant lesion. Overall, spectroscopy reflected the ultimate pathology of a low-grade, IDH-mutant oligodendroglioma. In contrast, there were no convincing findings indicative of an oligodendroglioma on structural imaging, with MRS at 7T providing additional diagnostic information.

More accurate pre-operative characterisation of glial lesions aids a more informed decision-making process for patients, surgeons, and oncologists alike. It can provide reassurance that a more conservative follow-up approach is acceptable until there is evidence of change. It may help in planning excision margins and the need for an awake craniotomy.

MRS at 7T is promising in non-invasively establishing the biochemical and molecular profile of gliomas, which could aid pre-operative and treatment planning, including targeting the most informative areas for biopsies, defining volumes of resection, and improving target volumes for radiotherapy. 7T MRS enables enhanced spectral dispersion and resolution of overlapping resonances and metabolites, with reduced Cramér-Rao lower bound (CRLB) values (McCarthy et al., 2022b). This is leading to improved visualisation of metabolites related to tumour metabolism, including tNAA/tCho ratios, the presence of lactate and lipid, and the presence of Glutamate (Glu), Glutamine (Gln), Glutathione (GSH), Glycine (Gly), and 2-hydroxyglutarate (2HG) (Andronesi et al., 2018), which are hypothesised to be of relevance for IDH prediction (Cadrien et al., 2024). MRS may require optimisation for different metabolites, including 2HG (Ganji et al., 2017). The increased importance of metabolic alterations in the 2021 tumour classification leads to an increased need to identify relevant metabolites pre-operatively, with 7T MRS offering this opportunity. To elevate 7T MRS into routine clinical practice, larger clinical studies and the creation of imaging databases are required, along with AI algorithms to generate improved predictive models based on metabolite concentrations, to pre-operatively predict tumour grade and molecular subgrouping, including IDH status, confidently and easily. Depending on the metabolites required to be measured and the clinical question to be answered, the prescribed echo time may need to be adjusted. The spectra demonstrated here were performed at short echo times to capture a wide range of metabolites, with the presence of 2HG also suggested, but not reliably measured, due to high CRLB values. For focused, optimised quantification of 2HG, a longer TE would be chosen (Emir et al., 2016).

Chemical Shift Imaging (CSI) of brain tumours, in addition to single voxel MRS, can be used to provide spatial, as well as metabolic, information. CSI, while better suited to assess heterogeneity within a tumour and examine areas surrounding the tumour for infiltration (Klauser et al., 2021), may struggle to provide the same resolution of metabolites within acceptable time limits for scanning and without increasing SAR levels.

In addition to the three cases of gliomas discussed above, three further post-operative cases are shown in Figures 5–10. These illustrate the difficulties in obtaining high-quality spectra in the presence of metal hardware, requiring adjustment of TRs and monitoring of SAR levels, which affect scanning times and the diagnostic quality of spectra. We have demonstrated that, with the application of appropriate safety protocols, spectra of high quality can be obtained at 7T.

The spectrum shown in Figure 6, which displays early reversal of normal tNAA/tCho and tCho/tCr ratios and a small lactate peak, supports the suspicion of an underlying neoplasm despite non-conclusive biopsies, with some features raising suspicion of an intermediate to higher-grade malignancy.

Early identification of patients at risk of recurrence post radio-chemotherapy is important for making early management decisions. Whilst calculation of power deposition in patients with post-operative metalwork can be difficult, adjustment of SAR limits and imaging parameters makes it possible to obtain high-quality data for early identification of metabolite alterations, as demonstrated in Figures 7, 8, which could suggest early disease progression. There may be a role for CSI here to cover larger areas of enhancing and non-enhancing abnormal white matter adjacent to the resection cavity and in the radiation field. Smaller-scale studies on routine field strength imaging have examined possible relationships between metabolite ratios and survival (Tolia et al., 2015). However, there appears to be little information available on high-field systems. The preliminary data presented in Figures 7, 8 show the potential of MRS at 7T in predicting early radiotherapy response and recurrence. Post-operative metal hardware should not be an impediment to using the capabilities of 7T MRI systems clinically in post-operative patients.

Interpretation of brain imaging post radiotherapy for high-grade gliomas can be difficult. Post-treatment effects, in particular radiation necrosis in patients with gliomas, especially glioblastomas, present a radiological dilemma (Sundgren, 2009). Radiation necrosis and pseudo-progression are relatively common occurrences after intracranial radiotherapy (Nichelli and Casagranda, 2021), particularly with newer chemotherapy agents and radiotherapy regimes, including the use of radiosensitisers. New or progressively enhancing lesions post-radiotherapy cause concern for patients and treatment dilemmas for clinicians. Routine imaging techniques frequently cannot resolve this dilemma, with MRS playing a potential role (Zeng et al., 2007). The potential role of 7T MRS in the differential diagnosis of radiation necrosis should be considered, as should the role of radiomics in identifying suitable imaging biomarkers for radiation necrosis.

The spectra presented in Figure 8 showed small lactate and lipid peaks, in addition to a decrease in the NAA peak and alteration of tNAA/tCho ratios, indicating a reduction in healthy neurons and loss of neuronal integrity, combined with an underlying anaerobic metabolic process. Whilst this may reflect residual/recurrent tumour, it may also indicate a degree of radiation necrosis (with the associated lactate peak) or a combination of both. In conjunction with the structural changes and increase in enhancement and brain permeability, this raised the possibility of early tumour progression. In contrast, the spectrum in Figure 10 showed no significant alteration in tNAA/tCho and tNAA/tCr peaks, with only slight lactate and lipid peaks present, favouring, in combination with the lack of significant progression of post-gadolinium enhancement in structural imaging, a degree of radiation necrosis over early tumour recurrence.

Spectroscopy at 7T offers several potential benefits over 3T MRS for clinical decision-making due to higher SNR and spectral resolution, allowing better dispersion of metabolite resonances that are overlapping and difficult to appreciate at 3T. The benefits of MRS at 7T are further enhanced by the high-resolution structural imaging of the tumour matrix. Pre-operative distinction of oligodendrogliomas from astrocytomas is facilitated by spectral separation of the Glx peak into Gln and Glu, and identification of IDH mutations at 7T enabled by improved detection and quantification of 2HG, which has the potential to affect decision-making regarding the timing and extent of surgery, as well as to allow non-invasive follow-up of patients by detecting changes in metabolite ratios early (Prener et al., 2023).

Identification of 2HG potentially heralds a new area in pre-operative tumour imaging, not yet fully exploited due to the difficulties in identifying this oncometabolite at lower field strengths and the continuing need to optimise MRS at higher field strengths for reliable quantification. The presence of 2HG is highly specific for IDH mutant gliomas (Han et al., 2020; Thomas et al., 2023). IDH 1 and 2 mutant gliomas have a more favourable prognosis compared to IDH wild-type gliomas, impacting treatment response and survival. 7T MRS for 2HG enables a non-invasive, virtual biopsy of a glioma, which aids in pre-operative planning and decision-making. Serial monitoring of 2HG can help detect progression and transformation non-invasively. Novel targeted IDH inhibitors (Cloughesy et al., 2025; Mellinghoff et al., 2023) are being assessed in clinical trials and have been licensed in several countries for the treatment of IDH mutant gliomas, with 2HG MRS facilitating monitoring of treatment responses.

The potential role of 7T MRI in MS (Bruschi et al., 2020; Fullerton et al., 2024; Louapre and Beigneux, 2021) is well recognised, demonstrating cortical lesions (Barkhof et al., 2025) that are not usually appreciable at lower field strengths; and demonstrating central vein signs (CVS) and paramagnetic rim lesions (PRLs) best seen at UHF, which are particularly relevant for the Revised 2024 McDonald Diagnostic Criteria (Barkhof et al., 2025; Martire et al., 2022; Montalban et al., 2025). On occasion, MS may present as a tumefactive demyelinating lesion (Al Malik, 2024; Algahtani et al., 2017), which can mimic a tumour, particularly if solitary, and cause diagnostic uncertainty, as demonstrated in Figure 11. One characteristic feature of TDLs is the presence of a glutamate peak on MRS, which is difficult to identify at lower field strengths (Cianfoni et al., 2007; Fullerton et al., 2024). UHF MRS should be considered as a diagnostic aid in cases where a TDL is causing diagnostic uncertainty. The ability to identify and separate metabolites at 7T can influence clinical decision-making regarding the nature of the lesion under investigation and can help avoid unnecessary biopsies and support decisions to start treatment promptly.

7T magnetic resonance, including both structural MRI and MRS, may be a valuable diagnostic aid for the early detection of suspected cases of PML. PML is caused by the reactivation of the John Cunningham Virus (JCV) and the infection of glial cells, including oligodendrocytes, leading to a potentially fatal demyelinating syndrome. Following a primary infection, JCV remains latent in renal and lymphoid tissue in immunocompetent hosts. Highly efficacious immunomodulatory treatments for MS can cause the virus to reactivate and damage the oligodendrocytes in the brain (Major et al., 2018; Wattjes et al., 2013). Timely imaging-based diagnosis (Wattjes and Barkhof, 2014; Williamson and Berger, 2017; Yousry et al., 2012) can be critical to avoiding debilitating morbidity or even fatality. While MRS by itself is unlikely to provide a diagnosis of PML, it could be an important clinical adjunct in the diagnosis of PML, which should be utilised, with a lactate peak (Kozic et al., 2017) often found.

MND is a spectrum of neurodegenerative disorders leading to progressive muscle weakness and ultimately death secondary to respiratory failure. MND is characterised pathologically by the loss of motor neurons in the motor cortex and anterior horn cells of the spinal cord. Different subtypes of MND, such as amyotrophic lateral sclerosis (ALS) and primary lateral sclerosis (PLS), have different clinical courses. The development of MND is likely multifactorial, with some genetic associations identified, including SOD1 and C9orf72 gene mutations (Dharmadasa et al., 2022), in addition to protein misfolding impacting proteostatic mechanisms.

There is a need for improved diagnostic approaches and the identification of non-invasive imaging biomarkers to diagnose and stratify MND earlier (Zejlon et al., 2024). The intrinsic properties of 7T make it an ideal tool for establishing imaging biomarkers for MND (De Vries et al., 2025). Identifying non-invasive imaging biomarkers could facilitate diagnostic pathways for patients and enable earlier stratification. Detecting microstructural degenerative changes in the motor cortex of patients with this disorder early in the disease process would increase diagnostic certainty. It is hypothesised that neurodegeneration is accompanied by alterations in the metabolic spectrum over the motor cortex, which are detectable using MR spectroscopy (Cheong et al., 2017; Kalra et al., 2006). Spectroscopy at 7T benefits from increased SNR and spectral resolution compared to lower field strengths. This allows for the robust quantification of metabolite peaks in the motor cortex, which may overlap at 3T. Consequently, the identification of novel metabolite biomarkers specific to subtypes of MND becomes possible. Glutathione, an antioxidant, is a potential marker of oxidative damage, which is hypothesised to be involved in the pathogenesis of MND (Kim, 2021). Alterations in tNAA levels indicate degeneration of healthy neurons in the motor cortex. Other metabolites that can be measured and separated at UHF, and are potentially relevant to neurodegeneration in ALS (Kalra, 2019), include inositol (Ins), a potential glial marker, glutamate, an excitatory neurotransmitter (which is difficult to separate from glutamine at lower field strengths), and GABA, an inhibitory neurotransmitter whose relative levels could be studied in MND using this technology. This is of particular interest as enhanced levels of neuronal excitability are observed in MND (Vucic et al., 2008).

We have demonstrated the feasibility of obtaining high-quality spectra at 7T over the affected motor cortex, with alterations in tNAA and tCho peaks and ratios, and inositol levels identified, as demonstrated. Furthermore, a small glutathione peak was evident. These findings are currently being further assessed in a larger study involving patients with different subtypes of MND, alongside the development of imaging biomarkers at 7T. MRS at 7T offers the possibility of detecting metabolites involved in the pathogenesis of different subtypes of MND, such as glutathione, an important antioxidant that is difficult to separate from Glx and GABA peaks at 3T due to the lesser resolution. The identification of glutathione has the potential to stratify MND patients into different groups, target precision medicine therapeutic approaches, and monitor progression in serial measurements, with the potential to influence clinical decision-making.

Epilepsy is one of the most common chronic neurological disorders worldwide, with patients often experiencing co-morbidities and a shortened life expectancy. While medical treatment options for epilepsy have significantly increased over the last decade, approximately 30% of patients fail to become seizure-free (Gueryi and Rheims, 2021; Perucca et al., 2023). For these patients with drug-resistant epilepsy, surgery is often the only option to achieve seizure freedom. Clinical assessments and tests include prolonged electroencephalography (EEG), seizure semiology, functional nuclear medicine imaging—including interictal ^18^F FDG PET, ictal and interictal ^99m^Tc HMPAO SPECT—and structural imaging with 3T MRI. However, these approaches often fail to demonstrate an epileptogenic focus, which is a potential target for surgery. Identification of an epileptogenic lesion or seizure onset zone is linked to a successful surgical outcome. With the improved resolution and enhanced contrast of 7T MRI, it is possible to detect epileptogenic structural abnormalities in patients previously diagnosed with “non-lesional” epilepsy, thereby facilitating stereotactic depth electrode implantations to define the seizure onset zone and enable curative surgery (De Ciantis et al., 2016; Opheim et al., 2021; Sharma et al., 2021; Rondinoni et al., 2021).

Temporal lobe epilepsy is a common focal epilepsy subtype. A well-recognised “lesional” abnormality is mesial temporal sclerosis (MTS). However, it is frequently challenging to localise or lateralise the seizure onset zone in temporal lobe epilepsy. While 7T MRI of the brain generally benefits from high-resolution imaging, the temporal lobes are often poorly visualised due to inhomogeneity and signal loss in that region caused by the shorter RF wavelength at 7T. Imaging of the temporal lobes at UHF remains challenging, including spectroscopy. The example of a spectrum in temporal lobe epilepsy with amygdala enlargement demonstrates that high-quality spectra can be obtained at 7T at the skull base, even within the anteroinferior temporal lobe, with appropriate expertise, shimming, and optimisation.

MRS plays a role in temporal lobe epilepsy by characterising any structural abnormalities and lesions detected, with hamartomatous and low-grade glial lesions sometimes presenting with similar imaging features. However, MRS may also play an important role in aiding the lateralisation of seizure onset zones in epilepsy (Castillo et al., 2001; Simister et al., 2002), assisting with pre-operative planning for both depth electrode insertion and resection. The involvement of neuronal networks in temporal lobe epilepsy makes the localisation and lateralisation of seizure onset and adjacent tracts increasingly important. Seizures are often associated with altered metabolites and neurotransmitters, including increased levels of excitatory neurotransmitters such as glutamate, which can be better differentiated and separated at UHF than in routine clinical 3T MRS. Dysfunction of glutamate-glutamine-GABA cycling (Eid et al., 2008; Pan et al., 2008) is thought to play an important role in epilepsy, with these neurotransmitters being difficult to resolve at routine clinical field strength. The example shown of the patient with temporal lobe epilepsy and amygdala enlargement (Figure 14) demonstrates a reduction in the tNAA and inositol peaks, as has been described in the literature, but low Glu and no Gln, indicating no evidence of upregulation of glutaminergic activity. The full potential of MRS at 7T in patients with temporal lobe epilepsy to assist in lesion characterisation, seizure onset lateralisation, and understanding of seizure aetiology and pathology has not yet been fully explored clinically (Perucca et al., 2023). We are currently conducting a study to further evaluate its role in temporal lobe epilepsy and the potential inclusion of MRS into clinical 7T protocols. Clinically, the identification and delineation of seizure onset zones are crucial for any potential epilepsy surgery. 7T MRS enables the identification of metabolites and neurotransmitters involved in seizure generation, which cannot be separated at 3T spectra. This is a potentially powerful clinical adjunct, guiding depth electrode placement and ultimately surgical decision-making.

With increased spectral resolution at higher field strengths, it is now possible to separate metabolites with structural similarity and resulting spectral overlap. The ability to separate the amino acids glutamate (Glu) and glutamine (Gln) at 7T ^1^H MRS is one of the strengths that spectroscopy at UHF has to offer. The chemical structures of Glu and Gln are similar, differing only in that Glu has a carboxylic acid and Gln has an amide side chain, leading to spectral overlap, with Glu resonating at 2.35 ppm and Gln at 2.45 ppm. MRS at lower field strengths frequently fails to adequately separate these peaks, which have been represented as a so-called “glx” peak in lower field strength spectroscopy (Ramadan et al., 2013). There is increasing awareness of the role of the neurochemicals Glu and Gln, and Glu-Gln cycling, in the brain, leading to heightened interest in separating these peaks in spectroscopy (Bell et al., 2025). Glu is the major excitatory neurotransmitter in the brain, with altered concentrations in many pathologies (Cai et al., 2012; Ramadan et al., 2013), including tumefactive demyelinating lesions, glioma, MND, and epilepsy, as discussed and demonstrated in this article, as well as traumatic brain injury, hepatic encephalopathy, Alzheimer’s, and dementia, amongst others (Abdelhamid et al., 2022; Fayed et al., 2011; Mao et al., 2019). This illustrates how MRS at 7T enables the identification and separation of neurochemicals and neurotransmitters; the quantification of Glu and Gln can serve not only as an imaging biomarker in the differentiation of astrocytomas from oligodendrogliomas and for TDLs, but also as a marker of metabolism, helping us better understand the pathogenesis of disorders such as epilepsy. The importance of separating Glu from Gln, made possible by 7T ^1^H MRS, in gliomas goes further than simply differentiating astrocytomas from oligodendrogliomas. Glu is also speculated to play an important role in high-grade glioma metabolism, and its identification and separation from Gln offer insight into metabolic glioma pathways and the possible role of glutamatergic synapses in tumour progression (Hangel et al., 2019; McCarthy et al., 2022a; Venkataramani et al., 2019). For patients with MND, the possibility to spectrally separate Glu from Gln at UHF and understanding Glu metabolism could provide valuable insight into disruption of the Glu/GABA balance and Glu/Gln cycle, and therefore pathogenesis of MND in addition to Glu functioning as an imaging biomarker.

We have shown the potential value of MRS at UHF for a variety of neuropathological conditions, with metabolites measured that are difficult to resolve at lower field strengths. MRS should always be interpreted in combination with structural imaging and in a clinical context. The combination of high resolution at 7T, high SNR, and improved susceptibility contrast, together with the increased spectral resolution, makes MRS at UHF a potentially powerful diagnostic tool.

We have highlighted metabolites and metabolite alterations associated with different glioma subtypes, grades, and mutations, and have indicated possible clinical applications, both for pre-operative and pre-treatment patients, to resolve clinical dilemmas. It should be noted that MRS can be successfully undertaken at 7T in more difficult anatomical locations, such as the temporal lobes, and within the SAR restrictions imposed on post-operative patients.

## 5 Conclusion

In this work, we have discussed the technical aspects and wide clinical potential of ^1^H MRS at 7T, which is not yet fully exploited. Technical development and optimisation have opened the door for the clinical use of MRS at UHF with the development of novel imaging biomarkers for a range of neurological pathologies. Further validation in larger studies is required to determine whether 7T MRS can reveal disease-specific metabolic signatures, improving diagnostic accuracy and informing patient management across diverse neurological conditions. This will enable its incorporation into “routine” imaging at 7T and the treatment of patients, of increasing relevance with the expansion of 7T sites worldwide.

## Data Availability

The datasets presented in this article are not readily available because they are patient data. Requests to access the datasets should be directed to Natasha Fullerton, natasha.fullerton2@nhs.scot.
